# NK cell expansion requires HuR and mediates control of solid tumors and long-term virus infection

**DOI:** 10.1084/jem.20231154

**Published:** 2023-09-12

**Authors:** Sytse J. Piersma, Sushant Bangru, Jeesang Yoon, Tom W. Liu, Liping Yang, Chyi-Song Hsieh, Beatrice Plougastel-Douglas, Auinash Kalsotra, Wayne M. Yokoyama

**Affiliations:** 1 Division of Rheumatology, Department of Medicine, Washington University School of Medicine, St. Louis, MO, USA; 2 Siteman Cancer Center, Washington University School of Medicine, St. Louis, MO, USA; 3 Department of Biochemistry, University of Illinois Urbana-Champaign, Champaign, IL, USA; 4 Cancer Center at Illinois, University of Illinois Urbana-Champaign, Champaign, IL, USA; 5 Carl R. Woese Institute for Genomic Biology, University of Illinois Urbana-Champaign, Champaign, IL, USA; 6 Bursky Center for Human Immunology and Immunotherapy Programs, Washington University School of Medicine, St. Louis, MO, USA

## Abstract

Natural killer (NK) cells are lymphocytes capable of controlling tumors and virus infections through direct lysis and cytokine production. While both T and NK cells expand and accumulate in affected tissues, the role of NK cell expansion in tumor and viral control is not well understood. Here, we show that posttranscriptional regulation by the RNA-binding protein HuR is essential for NK cell expansion without negatively affecting effector functions. HuR-deficient NK cells displayed defects in the metaphase of the cell cycle, including decreased expression and alternative splicing of *Ska2*, a component of the spindle and kinetochore complex. HuR-dependent NK cell expansion contributed to long-term cytomegalovirus control and facilitated control of subcutaneous tumors but not tumor metastases in two independent tumor models. These results show that posttranscriptional regulation by HuR specifically affects NK cell expansion, which is required for the control of long-term virus infection and solid tumors, but not acute infection or tumor metastases, highlighting fundamental differences with antigen-specific T cell control.

## Teaser

Piersma et al. identify a crucial role for posttranscriptional regulation by HuR in NK cell proliferation. This study reveals that HuR-dependent NK cell expansion is required for control of long-term virus infection and solid tumors without affecting acute infection or tumor metastases.

## Graphical Abstract

**Figure:**
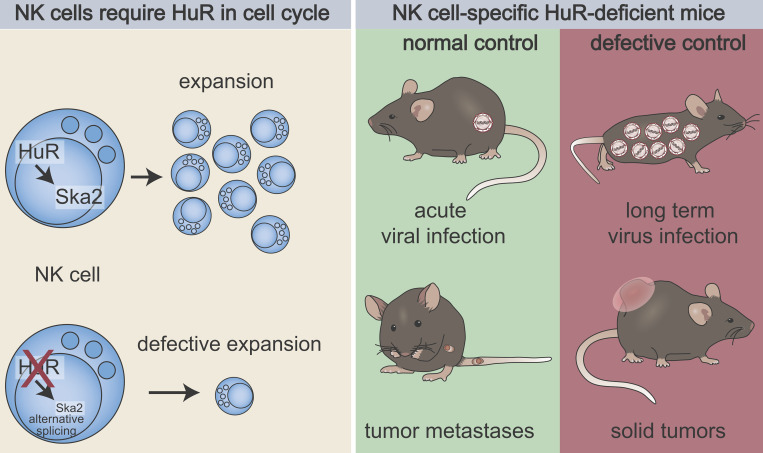

## Introduction

Natural killer (NK) cells are innate lymphoid cells (ILCs) capable of eliminating tumor and virus-infected cells through direct lysis and cytokine production (Piersma and Brizić, 2021). In response to viral infection, NK cells expand, contract, and form a long-lived compartment with increased effector functions analogous to adaptive lymphocytes (Mujal et al., 2021), yet studies on the role of NK cell expansion in tumor and viral control remain somewhat difficult to reconcile. In contrast to adaptive lymphocytes that utilize genetically rearranged receptors, NK cells recognize target cells based on a set of germline-encoded inhibitory and activation receptors. Even though the NK cell repertoire is diverse (Horowitz et al., 2013), it is estimated to be up to 10^4^-fold less diverse compared with the T cell repertoire (Qi et al., 2014). In contrast to rare naïve antigen-specific T cells that must undergo clonal expansion that takes many days to reach a critical mass to control infected cells or tumors, large numbers of virus-specific NK cells can respond at an earlier phase to control viral infections. Despite their early response to viral infections, NK cells expand in number (Dokun et al., 2001; Sun et al., 2009). For example, murine cytomegalovirus (MCMV) infection in C57BL/6 mice is controlled by Ly49H, an activation receptor expressed by ∼60% of naïve NK cells, which is responsible for genetic control of MCMV (Brown et al., 2001; Lee et al., 2001). Ly49H^+^ NK cells recognize the virus-encoded MHC class I (MHC-I) ortholog m157 and control MCMV beginning within 2–3 d (Parikh et al., 2015; Arase et al., 2002; Smith et al., 2002; Loh et al., 2005). Moreover, Ly49H-specific NK cell expansion starts later, around day 4, and peaks around day 7 post-infection (p.i.), and then the population contracts to form a long-lived compartment (Dokun et al., 2001; Sun et al., 2009). Thus, NK cell expansion occurs after the virus has been controlled, questioning the impact of NK cell expansion on control of acute virus infections.

While the role of NK cell expansion in control of solid tumors remains largely unexplored, studies of human patients, however, suggest that NK cell expansion may be critical. Patients who present with characteristics of NK cell deficiency, including a predisposition to virus infection and cancer, frequently have genetic defects in general cell division, including *MCM4*, *MCM10*, *GINS1*, and *RTEL1* (Gineau et al., 2012; Cottineau et al., 2017; Mace et al., 2020; Hanna et al., 2015; Mace and Orange, 2019). Since these inborn errors potentially point toward a critical role for NK cell expansion, the impact of NK cell proliferation on virus and tumor control requires further investigation.

NK cell proliferation can be regulated at the transcriptional level. For example, IL-12 signaling through STAT4 activates transcription programs required for NK cell expansion in response to MCMV infection (Sun et al., 2012). IRF8 is downstream of IL-12 and STAT4 signaling and is required for full NK cell expansion by increasing expression of the transcription factor Zbtb32 (Adams et al., 2018), which promotes NK cell proliferation by antagonizing the antiproliferative factor Blimp-1 (Beaulieu et al., 2014). In contrast, the transcription factor Fli1 represses NK cell expansion through increased levels of the proapoptotic factor Bim (Riggan et al., 2022), which is crucial in driving NK cell contraction after the Ly49H^+^ NK cell expansion phase (Min-Oo et al., 2014). Thus, the complex network of transcription factors that controls the NK cell response on a transcriptional level is emerging.

NK cell responses may be further regulated at the posttranscriptional level, such as regulation of RNA stability, initiation of translation, and splicing. These processes are affected by RNA-binding proteins that regulate key general and cell type–specific pathways in the immune system (Turner and Diaz-Muñoz, 2018). An important RNA-binding protein family in this context is the embryonic lethal, abnormal vision-like (*Elavl*) family encoding human antigen (Hu) proteins. *Elavl2–4* are mainly expressed in the brain, while *Elavl1* encoding HuR is more ubiquitously expressed (Mukherjee et al., 2011; Lebedeva et al., 2011). HuR mainly recognizes uridylate (U)- and adenylate-uridylate (AU)–rich sequences, primarily in the 3′ untranslated regions (3′UTR) of mRNAs but can also interact with intronic regions. HuR posttranscriptionally affects mRNA stability, translation, and mRNA splicing (Grammatikakis et al., 2017). HuR has selective functions within the hematopoietic system. It is required for postnatal hematopoiesis, myeloid and B cell development, but it does not affect CD4^+^ and CD8^+^ T cell numbers (Ghosh et al., 2009). In mature myeloid cells, HuR represses proinflammatory cytokine production and protects against LPS-induced endotoxemia (Yiakouvaki et al., 2012). B cells require HuR for metabolic fitness, which is required for an optimal antibody response (Diaz-Muñoz et al., 2015), while HuR is required for germinal center B cell maintenance and somatic hypermutation (Osma-Garcia et al., 2021). HuR negatively affects T cell maturation, deletion, and egress in thymocytes (Papadaki et al., 2009), whereas in mature CD4^+^ T cells, HuR affects cytokine production (Gubin et al., 2014). Thus, HuR mediates cell type– and stage-specific functions within the immune system, the impact of HuR on posttranscriptional regulation in NK cells is unknown, and its effect on cell proliferation in other lymphocytes has not been previously described.

Here, we report that HuR-dependent NK cell expansion is required for the control of long-term virus infections and solid tumors but not metastases. Mechanistically, NK cells require posttranscriptional regulation by HuR to facilitate expansion by regulating expression and splicing of cell cycle complexes in later stages of cell division including *Ska2*.

## Results

### NK cells specifically express ELAVL1 encoding HuR

**Figure 1.**
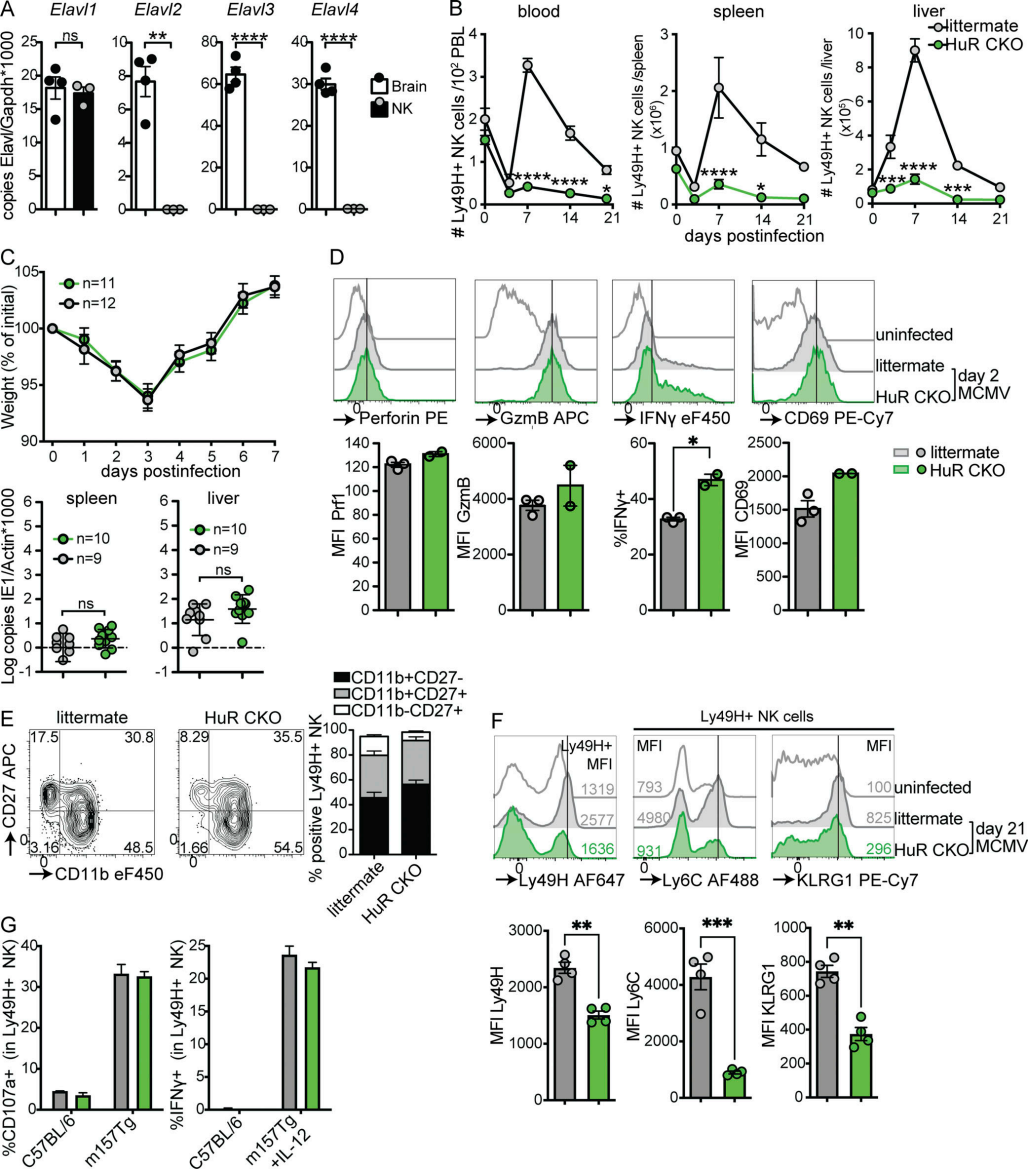
**NK cells specifically express Elavl1 encoding HuR that is required for expansion and formation of adaptive NK cells, but not effector functions in response to MCMV infection. (A)** Expression of *Elavl* family member transcripts in purified NK cells and brain. Statistics represent unpaired *t* tests with Bonferroni-Dunn correction from two independent experiments, totaling three to four mice per group. **(B)** Number of NK cells in blood, spleen, and liver in response to MCMV infection. Statistics indicate the comparison of HuR CKO with littermate NK cells at the indicated day after infection. PBL, peripheral blood leukocytes. Data are cumulative from six independent experiments totaling 4–11 mice per group per time point, with at least two independent experiments per time point. Statistics indicate two-way ANOVA with Bonferroni correction. **(C)** Weight loss and viral load in HuR CKO and littermate mice in response to MCMV infection. Viral load was measured by qPCR on day 5 p.i.; the dotted line indicates limit of detection. Cumulative data of two independent experiments for each panel totaling 9–12 mice per group. Statistics for weight loss were calculated using two-way ANOVA with Bonferroni correction, and for viral load unpaired *t* tests were used. **(D)** Expression of the effector molecules perforin, granzyme B (GzmB), IFNγ, and CD69 by splenic NK cells at 36 h after MCMV infection. Representative data from two independent experiments with two to three mice per group. Statistics are unpaired *t* tests with Bonferroni-Dunn correction. **(E)** Expression of maturation markers CD27 and CD11b on splenic NK cells at 5 d after MCMV infection. Representative data from two independent experiments with three to five mice per group. **(F)** Expression levels of adaptive NK cell markers Ly49H, Ly6C, and KLRG1 at day 21 p.i. by splenic NK cells. Representative data from two independent experiments with four mice per group. Unpaired *t* tests with Bonferroni-Dunn correction were used for statistics. **(G)** Degranulation measured by CD107a staining and IFNγ production by HuR-WT and HuR CKO NK cells in response to stimulation with C57BL/6 or m157-Tg cells in the presence of IL-12 where indicated. Representative of two independent experiments with duplicates. MFI, median fluorescent intensity. Error bars indicate SEM; ns, not significant; *P < 0.05, **P < 0.01, ***P < 0.001, and ****P < 0.0001.

**Figure S1.**
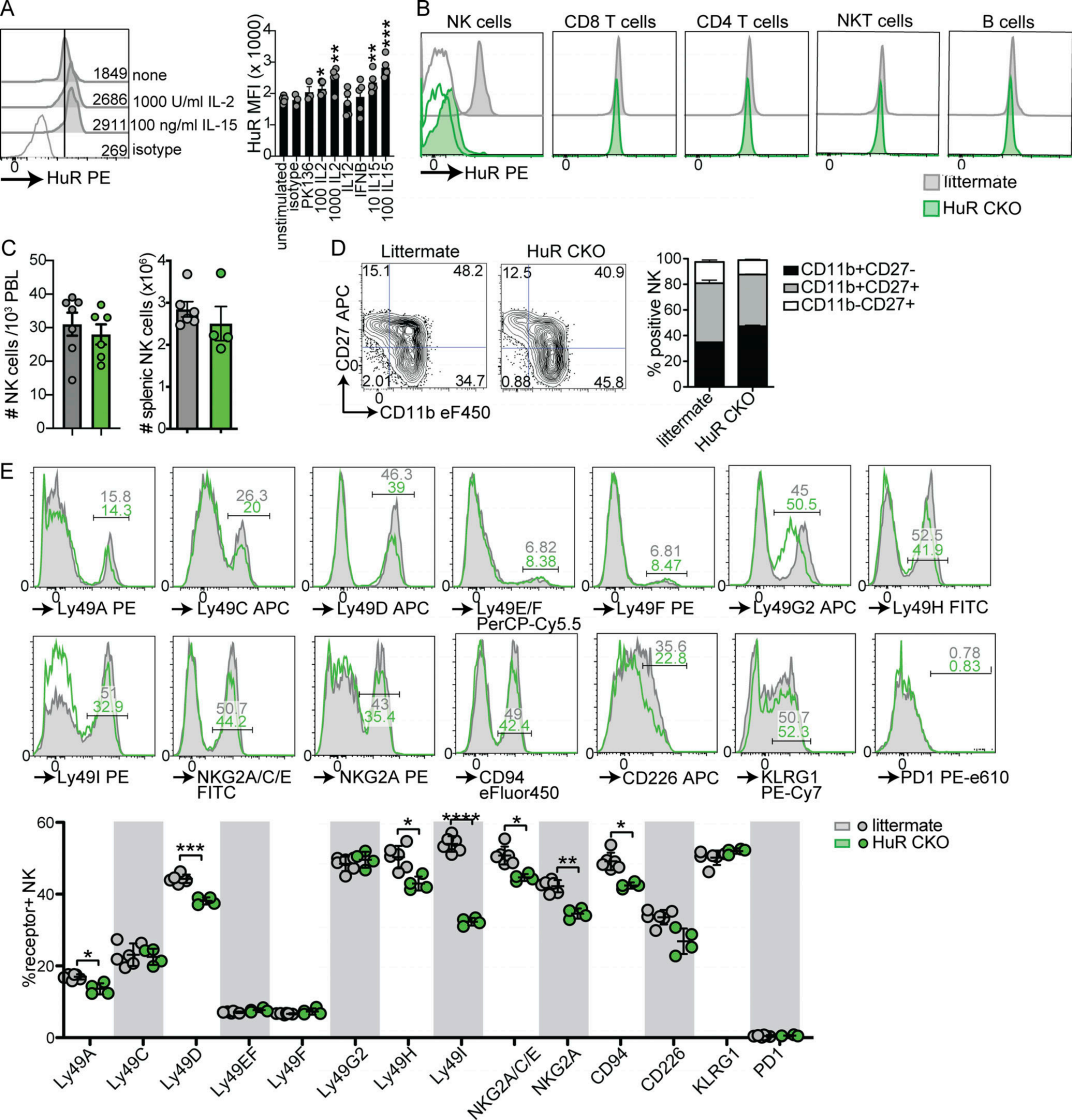
**HuR expression in NK cells and steady state phenotype of HuR CKO NK cells. (A)** Expression of HuR in NK cells in response to stimulation with plate-bound antibody or indicated cytokines for 1 d. Representative of two experiments in triplicate. **(B)** HuR expression by splenic lymphocytes in HuR CKO and littermate control mice. Filled histograms show HuR staining and open histograms indicate isotype control. Representative of two independent experiments. **(C)** Peripheral blood and splenic NK cell numbers in HuR CKO and littermates analyzed by flow cytometry. Cumulative of two experiments totaling four to six mice per group. **(D)** Maturation of splenic NK cells measured by CD27 and CD11b staining. **(E)** Receptor repertoire on splenic NK cells in HuR CKO and littermate control mice. Cumulative of two experiments totaling four to six mice per group. Statistics were calculated using unpaired *t* tests with Bonferroni correction. Error bars indicate SEM; ns: not significant, *P < 0.05, **P < 0.01, ***P < 0.001, and ****P < 0.0001. MFI, median fluorescent intensity.

We evaluated mRNA expression of *Elavl1–4* by quantitative PCR (qPCR) in NK cells compared with brain tissue that expresses all members (Fig. 1 A). *Elavl* encoding HuR was expressed constitutively in splenic NK cells (Fig. S1 A) at expression levels comparable with brain tissue, while the other family members were below detection limits. HuR levels increased in response to stimulation with IL-2 and IL-15, which promote proliferation and survival. However, triggering the NK1.1 activation receptor did not affect HuR levels (Fig. S1 A). Thus, NK cells specifically express HuR, and their constitutive expression is increased in response to proproliferative and prosurvival cytokines.

To investigate a potential role for HuR in NK cell function, we crossed *Elavl1*^*fl/fl*^ mice to *Ncr1*^*Cre*^ mice to obtain *Ncr1*^*Cre/wt*^ × *Elavl1*^*fl/fl*^ (HuR CKO) and *Ncr1*^*wt/wt*^ × *Elavl1*^*fl/fl*^ littermate controls to specifically target ILCs. While other ILCs may be affected, here we used experimental systems that have been previously attributed to NK cells. HuR CKO mice displayed efficient and specific deletion in the NK cell compartment, while other adaptive lymphocyte compartments were unaffected (Fig. S1 B). Total number of NK cells were similar between HuR CKO and littermate controls in peripheral blood and spleen (Fig. S1 C). We observed a modest increase in CD11b^+^CD27^−^ NK cell population in HuR CKO mice, indicative of an increased mature population (Fig. S1 D). HuR CKO NK cells expressed all NK cell receptors analyzed, albeit with some alterations in percentage of NK cells positive for Ly49D, Ly49H, Ly49I, NKG2A/CD94, and CD226 (Fig. S1 E). Thus, NK cell numbers and development were largely unaffected by HuR deficiency.

### HuR CKO mice acutely control MCMV but are deficient in NK cell expansion

**Figure S2.**
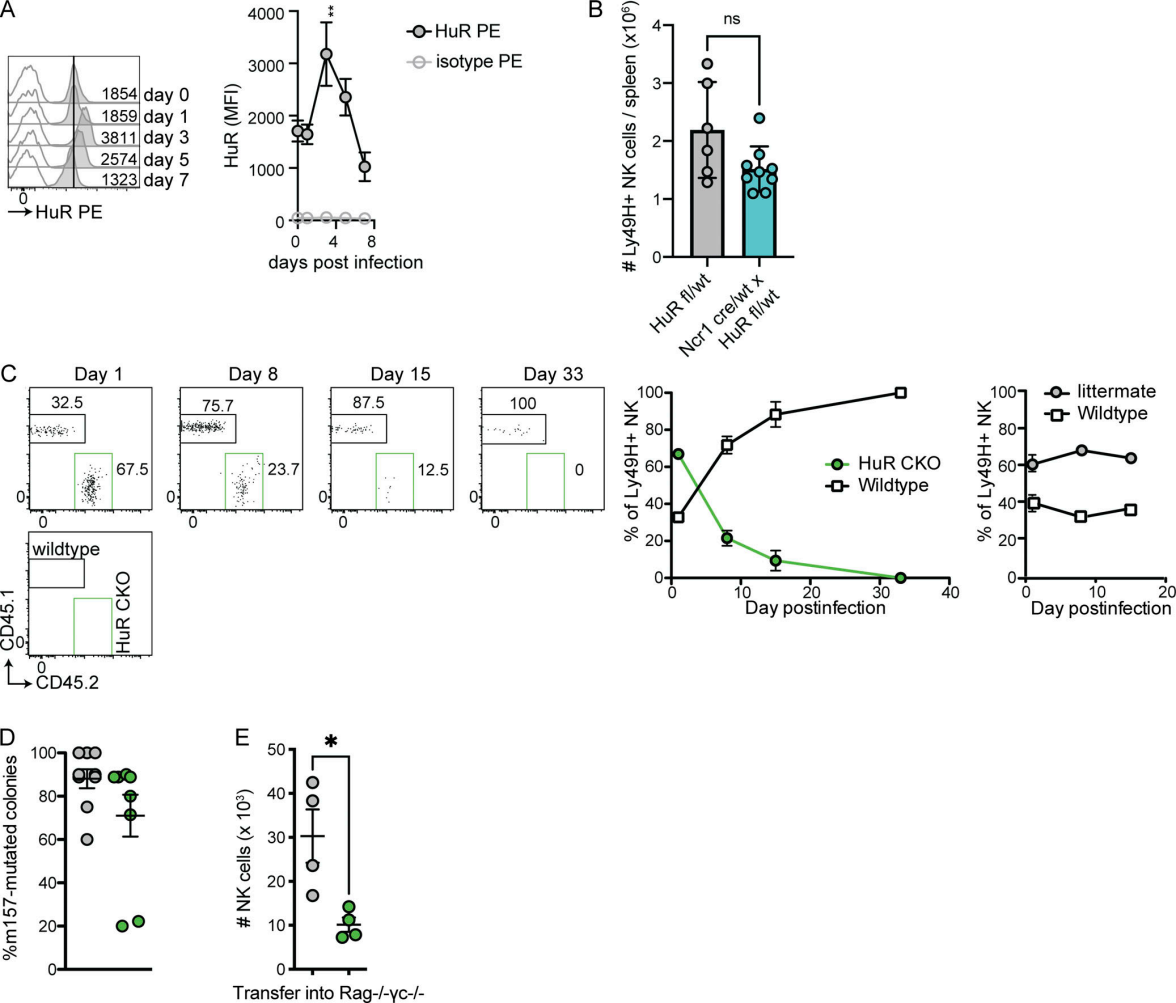
**HuR expression and function of HuR-deficient NK cells during MCMV infection. (A)** HuR expression by splenic C57BL/6 NK cells at indicated day after infection, analyzed by flow cytometry. Representative of two independent experiments with three mice per time point. **(B)** Number of splenic NK cells in Ncr1 cre/wt × HuR fl/wt and littermate Ncr1 wt/wt × HuR fl/wt control mice at 7 d after MCMV infection. **(C)** Competitive expansion of HuR CKO (left) or littermate control with congenic CD45.1 WT NK cells transferred into Ly49H-deficient hosts and subsequently infected with MCMV. Representative of two independent experiments with three to five mice per group. **(D)** Analysis of MCMV m157 sequences in spleens of RAG HuR CKO and RAG littermate mice that succumbed to MCMV infection. Cumulative of two independent experiments totaling nine mice per group with 10 m157 sequences analyzed per mouse. Statistics were calculated using unpaired *t* tests with Bonferroni correction. Error bars indicate SEM; ns: not significant, *P < 0.05, and **P < 0.01. MFI, median fluorescent intensity.

We exploited the well-established MCMV model to analyze the role of HuR in NK cell function in more detail. In response to MCMV infection, WT NK cells upregulated HuR expression that peaked at day 3 p.i. (Fig. S2 A), suggesting that HuR may affect NK cell responses to virus infection. MCMV infection causes Ly49H^+^ NK expansion, peaking at day 7 p.i., followed by a contraction phase and formation of a long-lived “adaptive” population (Sun et al., 2009; Dokun et al., 2001). Ly49H^+^ NK cell expansion was virtually absent in HuR CKO mice, while littermate controls displayed prototypic NK cell expansion and contraction in blood, spleen, and liver (Fig. 1 B). NK cells with heterozygous deletion for HuR (*Ncr1*^*Cre/wt*^ × *Elavl1*^*fl/wt*^) were able to expand to a similar level as littermate controls (Fig. S2 B), indicating that homozygous but not heterozygous deletion of *Elavl1* causes HuR dysfunction and that the observed phenotype is not caused by the *Ncr1*^*cre*^ allele.

Surprisingly, however, despite the lack of NK cell proliferation, morbidity and viral control were unaffected in HuR CKO mice up to day 7 (Fig. 1 C). Consistent with these observations, HuR CKO NK cells did not show defects in accumulation of the cytolytic molecules granzyme B and perforin, IFNγ production, and upregulation of the activation marker CD69 at day 1.5 p.i. (Fig. 1 D). HuR CKO NK cells did not show defects in virus-induced maturation at day 5 p.i. (Fig. 1 E). Formation of the Ly49H “adaptive” compartment is associated with increased Ly49H, Ly6C, and KLRG1 expression (Sun et al., 2009). This phenotype was absent in HuR-deficient NK cells at day 21 p.i., while littermate control NK cells displayed increased expression of Ly49H, Ly6C, and KLRG1 (Fig. 1 F). These data suggest that “adaptive” NK cells in the spleen were potentially replaced by naïve NK cells emigrating from the bone marrow in HuR CKO mice. In line with these observations, cotransfer of HuR CKO and congenically labeled WT NK cells into Ly49H-deficient hosts resulted in preferential outgrowth of WT NK cells, whereas littermate control NK cells were not outcompeted (Fig. S2 C). We were unable to detect any HuR CKO NK cells at day 33 p.i., again suggesting a defect in the formation of an “adaptive” Ly49H^+^ compartment in HuR CKO mice. Moreover, HuR CKO NK cells degranulated and produced IFNγ upon stimulation with m157-expressing target cells in vitro (Fig. 1 G). Collectively, these results show that HuR is required for NK cell expansion and formation of an adaptive NK cell compartment but is dispensable for NK cell effector functions and initial control of MCMV infection.

### HuR CKO mice have defective long-term viral control in the absence of adaptive immunity

**Figure 2.**
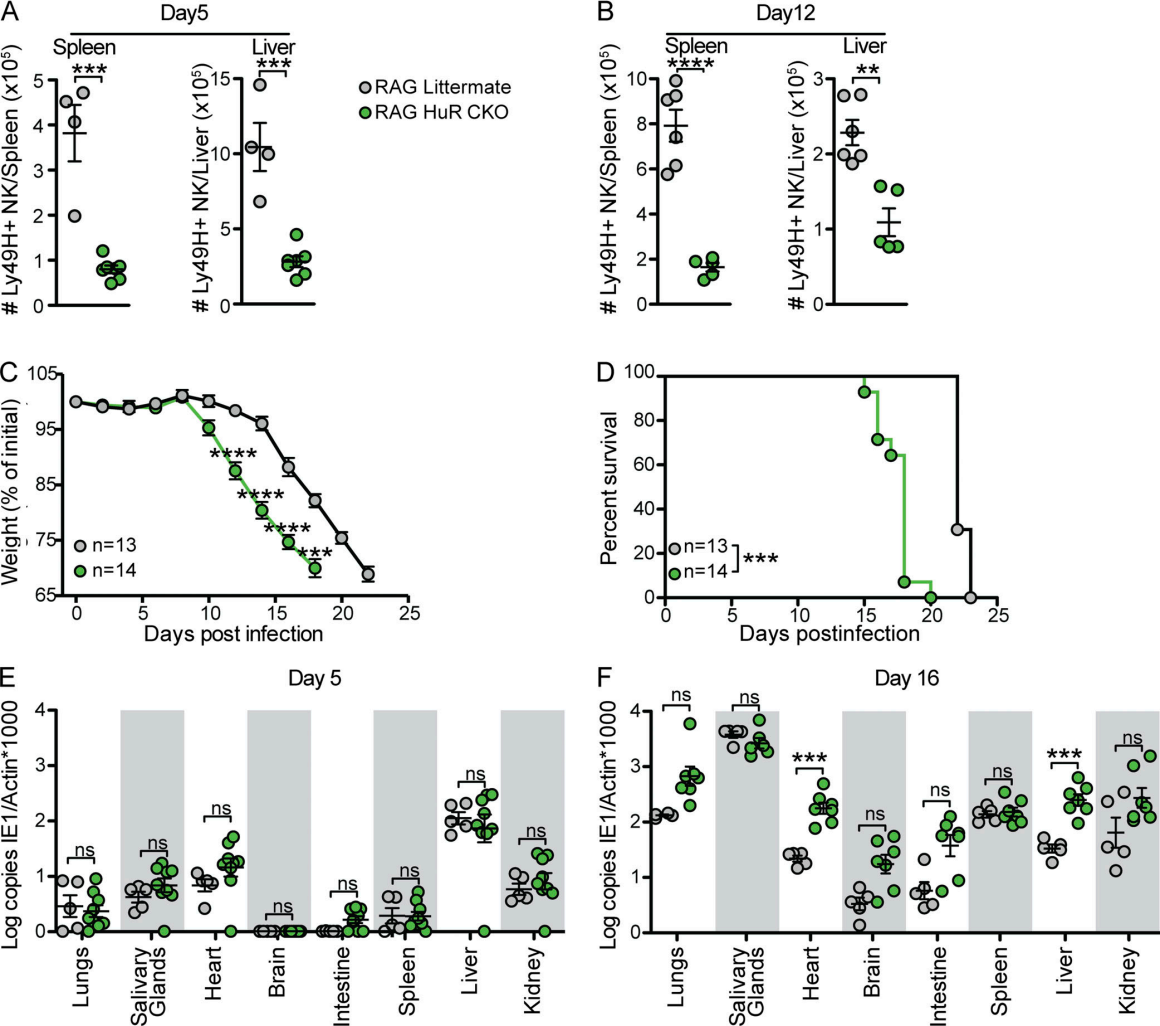
**Rag-deficient HuR CKO mice have increased organ-specific susceptibility to MCMV infection at later time points. (A and B)** Numbers of NK cells in spleen and liver at day 5 (A) and day 12 (B) p.i., with each panel representing an independent experiment with four to seven mice per group. Statistics represent unpaired *t* tests with Bonferroni-Dunn correction. **(C and D)** Weight loss (C) and survival (D) in RAG HuR CKO and RAG littermate mice after i.p. infection with MCMV. Cumulative data of three independent experiments totaling 13–14 mice per group. Statistics for weight loss were calculated using two-way ANOVA with Bonferroni correction and indicate a comparison of HuR CKO RAG1 with littermate RAG1 NK cells at the indicated day p.i. Survival statistics were calculated using Log-rank (Mantel-Cox) tests. **(E and F)** Viral load in indicated organs at day 5 (E) and day 16 (F) p.i. analyzed by qPCR. Each panel is cumulative data from two independent experiments totaling four to nine mice per group. Statistics were calculated with unpaired *t* tests with Bonferroni-Dunn correction. Error bars indicate SEM; ns, not significant; **P < 0.01, ***P < 0.001, and ****P < 0.0001.

As the expansion defect in HuR CKO NK cells precluded investigation of HuR CKO adaptive NK cell function and long-term viral control in adoptive transfer experiments, we generated HuR CKO mice on a RAG-deficient background to examine the long-term NK cell response to MCMV. Previous studies indicate RAG-deficient mice initially control MCMV, but the virus persists in the absence of adaptive immunity allowing the accumulation of m157 viral mutants, thereby escaping control by Ly49H^+^ NK cells, which causes the ultimate demise of the host around day 21–25 p.i. (French et al., 2004). Consistent with the defective HuR CKO NK cell expansion in RAG-sufficient mice, HuR CKO × RAG1 KO (RAG HuR CKO) mice displayed decreased numbers of Ly49H^+^ NK cells compared with littermate controls at day 5 and 12 p.i. in the spleen and liver (Fig. 2, A and B). Like RAG1-competent mice, MCMV-induced morbidity was unaffected in RAG HuR CKO mice in the first week p.i. (Fig. 2 C). Subsequently, as compared with HuR-sufficient, RAG-deficient littermate controls, RAG HuR CKO mice started losing weight at earlier time points, i.e., day 10 p.i., and they progressively lost weight until they succumbed to their infection, with median day 18 survival compared with day 22 for HuR-sufficient, RAG-deficient littermate controls (Fig. 2 D). Despite the accelerated disease progression, we detected the emergence of m157-deficient mutant viruses in the spleens of RAG HuR CKO mice (Fig. S2 D), which is due to Ly49H-dependent viral selection (French et al., 2004). Consistent with the observed morbidity, RAG HuR CKO mice had similar viral loads to RAG littermates in a wide range of organs at day 5 p.i. (Fig. 2 E) but had significantly increased viral loads at day 16 p.i. in the liver and heart (Fig. 2 F). Collectively, these data indicate that HuR is not required for NK cell–mediated control of acute MCMV infection but is required for long-term viral control.

### HuR-deficient NK cells fail to proliferate due to defects in late cell cycle stages

**Figure 3.**
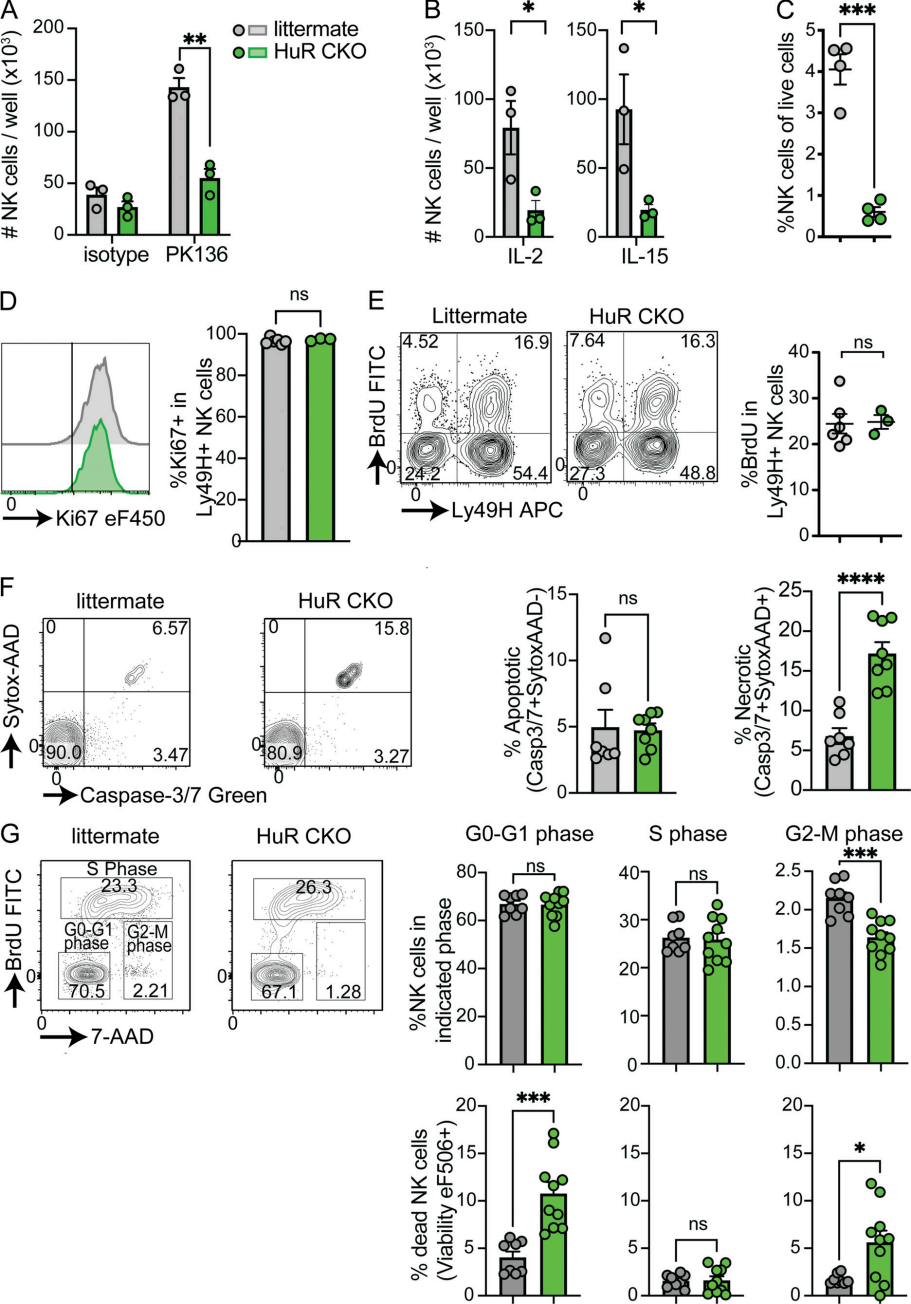
**Activated HuR-deficient NK cells have defective proliferation, resulting from increased cell death. (A)** Purified NK cells were stimulated with indicated plate-bound antibody in the presence of low-dose IL-2, and the number of NK cells was analyzed after 4 d by flow cytometry using counting beads. Representative of two independent experiments with three mice per group. **(B)** Purified HuR WT or HuR CKO NK cells were expanded with high-dose IL-2 or IL-15. Representative of two independent experiments with three mice per group. **(C)** Purified NK cells were transferred to *Rag2*^*−/−*^*Il2rg*^*−/−*^; 14 d after transfer, NK cells were quantified in the liver. Representative of two independent experiments with four mice per group. **(D and E)** Mice were infected with MCMV, and at day 5 p.i. mice were pulsed with BrDU i.p. for 3 h, after which NK cells were analyzed for Ki67 (D) and BrdU incorporation (E) in the spleen by flow cytometry. Representative of two independent experiments with three to five mice per group. **(F)** Mice were infected with MCMV, and after 3 d, NK cells were analyzed for apoptosis and cell death by flow cytometry. Cumulative of two independent experiments totaling seven to eight mice per group. **(G)** HuR WT or HuR CKO splenocytes were expanded with high-dose IL-2; after 4 d, cells were pulsed with BrDU for 1 h, after which cell cycle and viability in NK cells were analyzed by flow cytometry. Cumulative of two independent experiments totaling four to five mice per group in duplicate. Statistics were calculated with unpaired *t* tests. Error bars indicate SEM; ns, not significant; *P < 0.05, **P < 0.01, ***P < 0.001, and ****P < 0.0001.

The observed in vivo defects in NK cell accumulation in virus-infected HuR CKO mice pointed toward a potential defect in NK cell proliferation in HuR CKO mice. To investigate the proliferation defect without the influence of cell migration and other factors, activation receptor-dependent NK cell proliferation was assessed in vitro by stimulation with plate-bound anti-NK1.1 antibodies in the presence of low-dose IL-2, as previously described (Reichlin and Yokoyama, 1998). HuR CKO NK cells displayed defective proliferation in response to anti-NK1.1 compared with littermate control NK cells (Fig. 3 A). This defect in HuR CKO NK cells extended to non-specific proliferation in response to high dose IL-2 and IL-15 as well (Fig. 3 B). Moreover, transfer of HuR CKO NK cells into lymphopenic *Rag2*^*−/−*^*Il2rg*^*−/−*^ resulted in decreased homeostatic proliferation in vivo as compared with littermate control NK cells (Fig. 3 C). Despite the defects in proliferation, HuR CKO NK cells were able to enter the cell cycle, as the percentage of Ki67^+^ NK cells was not affected in splenic NK cells in HuR CKO mice (Fig. 3 D). Similar BrdU incorporation was also observed (Fig. 3 E), indicating that DNA replication was not affected by HuR. MCMV-induced NK cell apoptosis was not influenced by HuR, as percentages of Caspase3/7^+^ actinomycin D^*−*^ cells were similar between HuR CKO and littermate controls (Fig. 3 F). In contrast, the percentage of cell death (Caspase3/7^+^ actinomycin D^+^) was increased in HuR CKO mice (Fig. 3 F). Detailed analysis of the cell cycle revealed a significant decrease in the percentage of HuR-deficient NK cells in the G2/M phase (Fig. 3 G, upper panels). Moreover, we observed increased cell death in the G2/M and the G0/G1 phases, but no increased death in the S phase (Fig. 3 G, bottom panels). Taken together, these results suggest that HuR is required for NK cell proliferation particularly in the later cell stages of cell division, resulting in increased cell death in the G2/M and subsequent G0/G1 phases.

### HuR-deficient NK cells display defects in expression and splicing in cell cycle–associated genes

**Figure 4.**
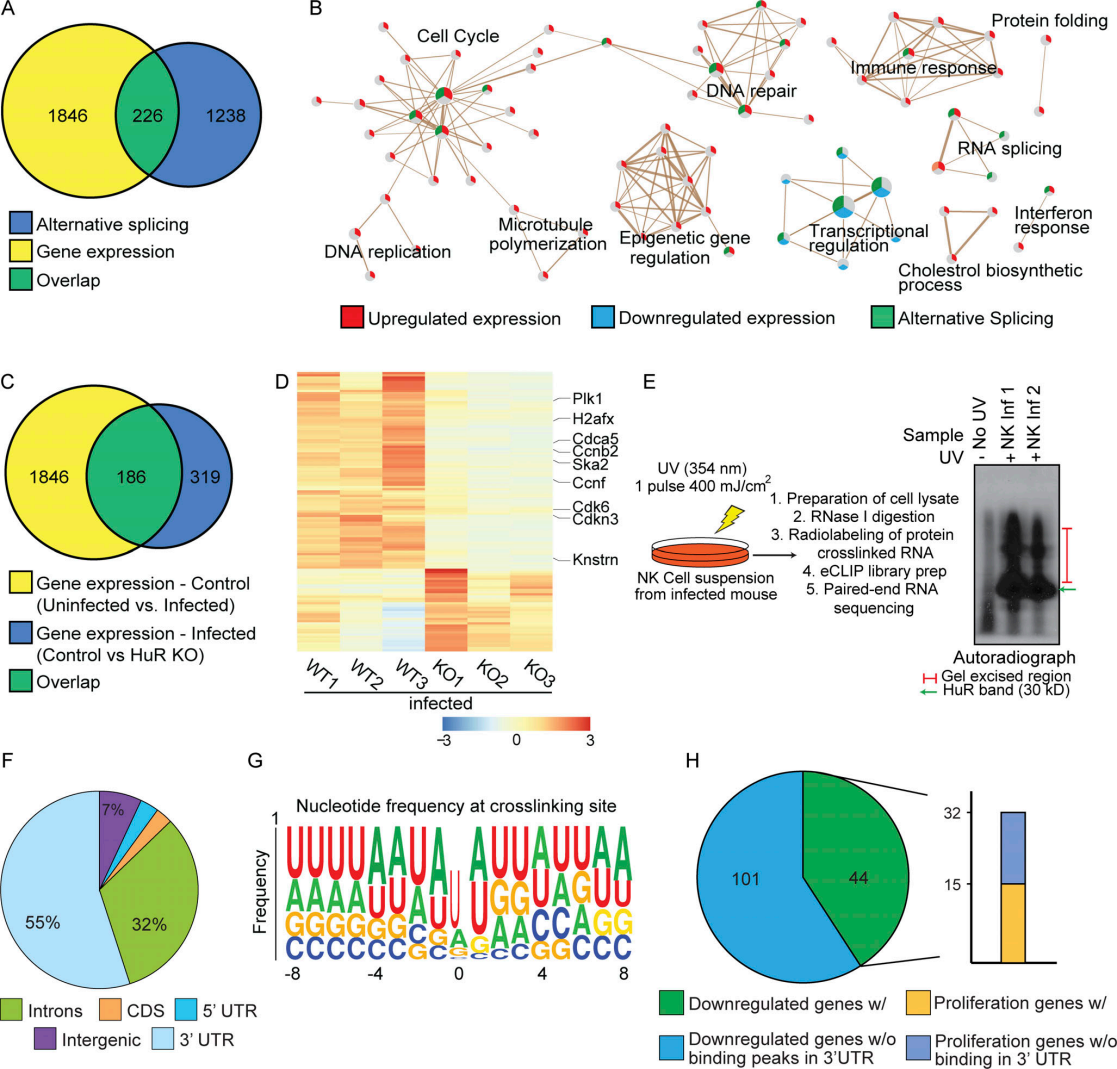
**Transcriptomic and eCLIP analysis of WT and HuR-deficient NK cells derived from MCMV-infected mice. (A and B)** Venn diagram (A) and gene ontology (B) of gene expression and alternative splicing changes on MCMV infection in littermate control NK cells. **(C)** Overlap of gene expression changes on MCMV infection in NK cells with changes due to loss of HuR in infected mice. **(D)** Heatmap showing a decrease in the expression of proliferation genes upon loss of HuR in MCMV infection. **(E)** Schematic for eCLIP experiment for HuR in NK cells from MCMV infection. **(F)** Distribution of HuR-binding peaks in the genome and across the gene body. **(G)** Frequency plot for nucleotide residues found at HuR crosslinking sites in the RNA. **(H)** Genes downregulated upon HuR loss in MCMV infection are found by HuR in 3′UTR, and among these about one half of the proliferation genes are also bound by HuR in 3′UTR regions.

Since HuR is an RNA binding protein that has been implicated in regulating posttranscriptional processes, including RNA stability and alternative splicing, we analyzed the transcriptome for mRNA expression and splicing in splenic NK cells from day 3 MCMV-infected HuR CKO and littermate control mice. RNA isolated from sorted NK cells was subjected to poly-A–selected RNA sequencing (RNA-seq). Upon comparing NK cells from infected control mice (WT-Infection) to uninfected mice (WT-Uninfected), we observed extensive gene expression and alternative splicing changes (Fig. 4 A). The genes upregulated in expression were specifically enriched for cell cycle pathways (Fig. 4 B), which is expected since NK cell numbers rapidly amplify after infection (Fig. 1 B).

In addition, alternatively spliced genes were also enriched for cell cycle–related pathways along with pathways involved in transcriptional regulation (Fig. 4 B). We found that ∼20% of genes that normally change in expression during infection in WT NK cells failed to change appropriately in HuR CKO NK cells, and these genes are specifically enriched for cell cycle pathway genes, potentially explaining the observed proliferation defects in NK cells upon loss of HuR (Fig. 4, C and D; and Fig. S5 A).

To understand the mechanistic cause of cell cycle defects in HuR CKO NK cells post-MCMV infection, we performed enhanced crosslinking and immunoprecipitation (eCLIP) of HuR in infected WT NK cells to identify the direct RNA-bound targets of HuR (Fig. 4 E). Consistent with previous studies of HuR in other cells, we found that the major binding preferences for HuR were intronic and 3′UTR of mRNAs in WT NK cells (Fig. 4 F). Additionally, crosslinking analysis of HuR-binding sites revealed a preference for AU-rich elements (Fig. 4 G and Fig. S5 B). We found that nearly one third of genes downregulated upon loss of HuR in MCMV infection were bound by HuR in WT NK cells in their 3′UTRs, and more specifically, about 50% of downregulated genes involved in cell cycle pathways were bound in 3′UTR regions by HuR (Fig. 4 H). Taken together, these data show that HuR-deficient NK cells display defects in expression and alternative splicing of genes associated with the cell cycle.

### Ska2 is aberrantly spliced and regulated in HuR-deficient NK cells

**Figure 5.**
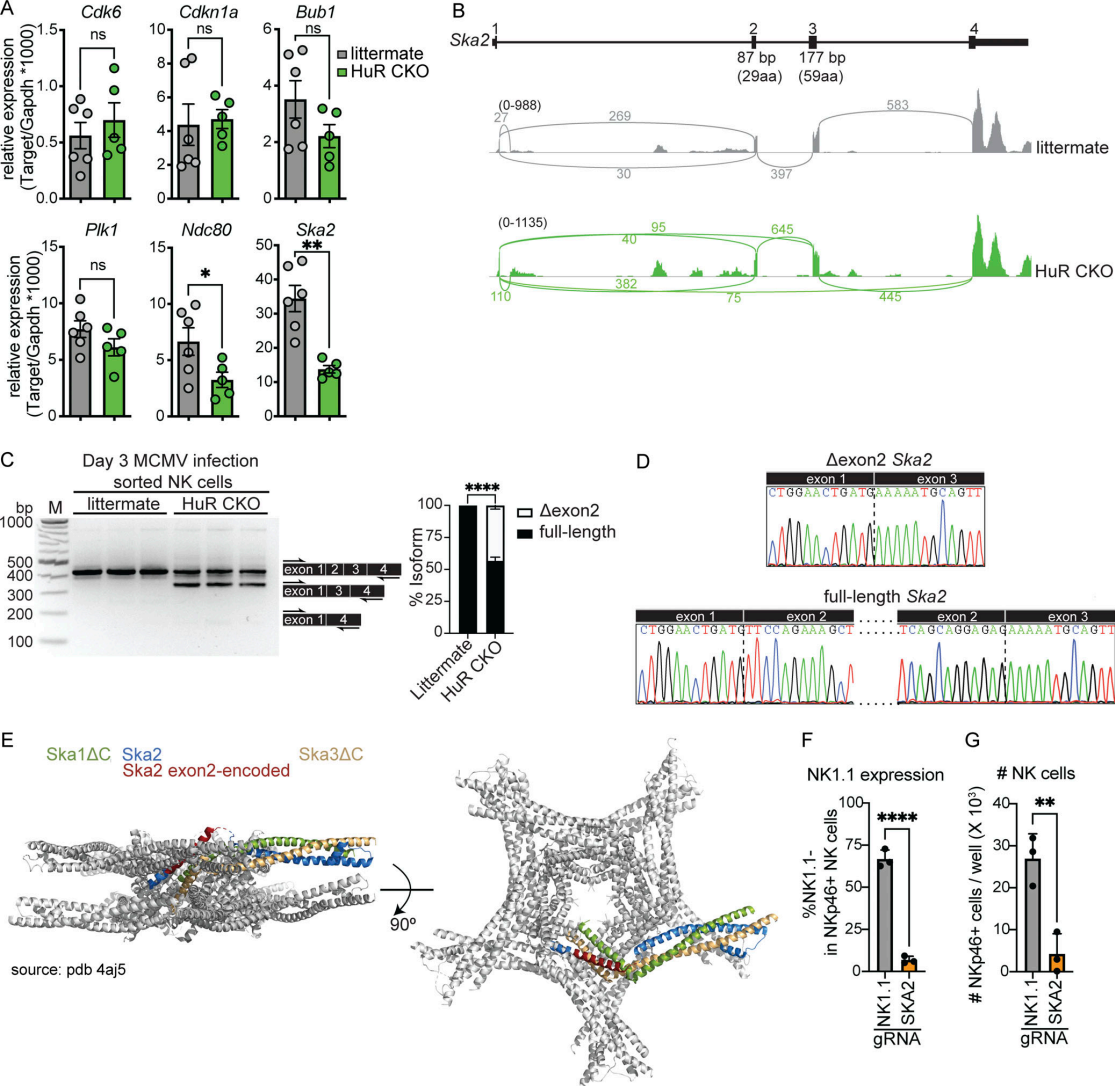
**Ska2 is aberrantly spliced in HuR-deficient NK cells, which causes decreased NK cell expansion. (A)** Splenic NK cells were sorted from day 3 MCMV-infected animals and relative mRNA copy number was analyzed by TaqMan qPCR. Cumulative data from two independent experiments totaling five to six mice per group. **(B)** Sashimi plot displaying Ska2 mRNA splicing in HuR CKO and littermate control NK cells. Sashimi plots are representative of the splicing dataset. **(C)** PCR along exon 1 to exon 4 of Ska2 to analyze alternative splicing in splenic NK cells isolated from day 3 MCMV-infected animals. The band intensity of different isoforms was analyzed using image lab software. Representative data from two independent experiments with three mice per group. M, marker. **(D)** The nucleotide sequences of gel-excised bands were analyzed by Sanger sequencing. **(E)** Cartoon representation of the structure of the Ska core complex using PDB 4aj5. Indicated in red is the portion of Ska2 encoded by exon 2 in 1 out of 10 Ska2 molecules within the Ska complex. **(F and G)** C57BL/6 splenocytes were electroporated with Cas9 and specific gRNAs and cultured with IL-15 for 4 d. NKp46^+^CD3^−^CD19^−^ NK cells were analyzed for NK1.1 expression (F) and cell number (G). Representative data from two independent experiments with three mice per group. Statistics were calculated with unpaired *t* tests. Error bars indicate SEM; ns, not significant; *P < 0.05, **P < 0.01, and ****P < 0.0001. Source data are available for this figure: SourceData F5.

**Figure 6.**
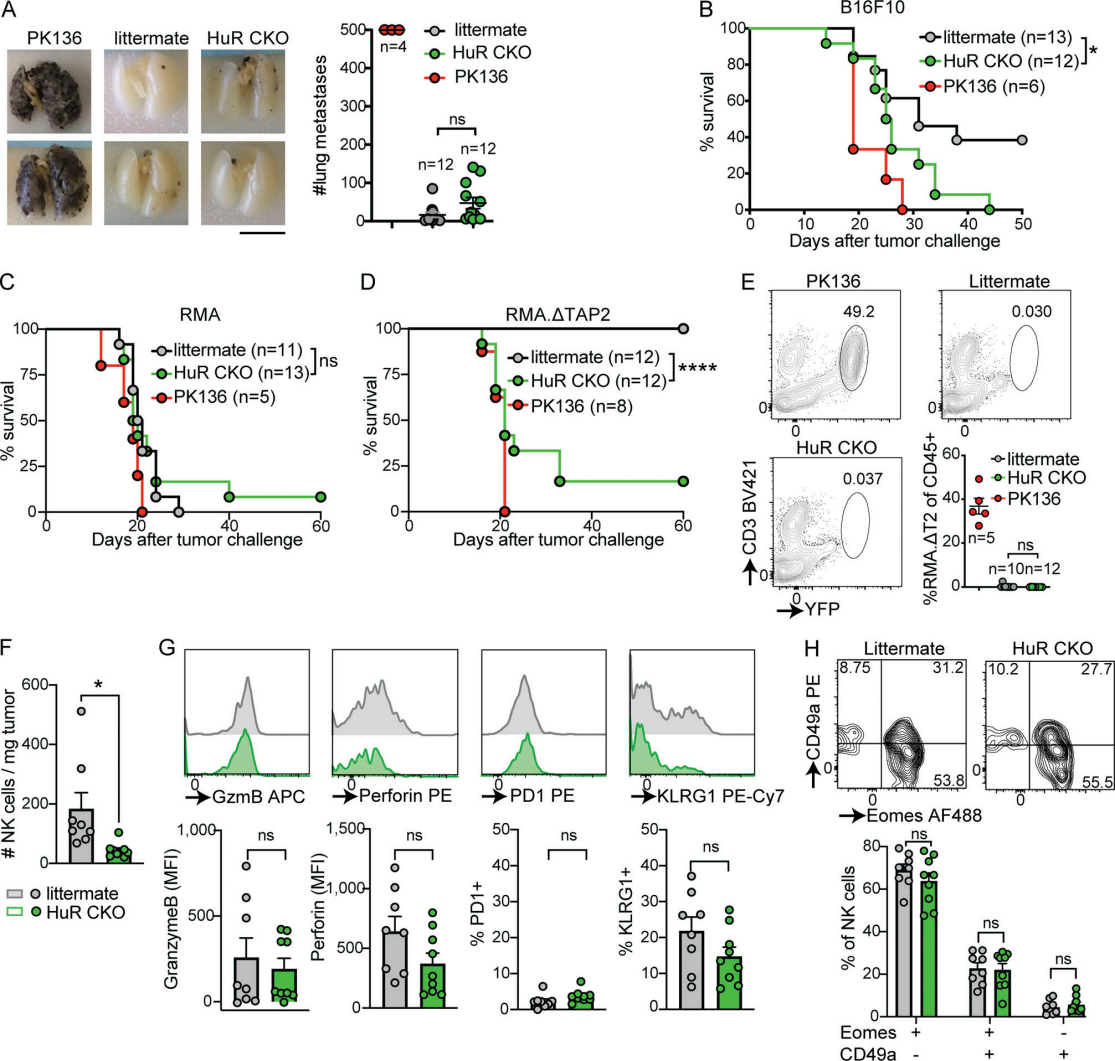
**HuR-dependent NK cell expansion is required for primary tumor control but is dispensable for elimination of tumor metastases. (A)** Mice were either untreated or NK cells were depleted with anti-NK1.1 (PK136). B16F10 melanoma cells were injected i.v. After 14 d, lungs were harvested and metastases were counted in a blinded manner. Scale bar indicates 10 mm. Cumulative of two independent experiments with 4–12 mice per group. Statistics represent unpaired *t* test. **(B)** B16F10 melanoma cells were injected s.c., and tumor growth was monitored; mice were sacrificed when tumor size was larger than 1,000 mm^3^. Mice were either untreated or NK cells were depleted with anti-NK1.1 (PK136). Survival of indicated groups is shown over time. Cumulative of two independent experiments with 6–13 mice per group and statistics are from Log-rank (Mantel-Cox) test. **(C and D)** MHC-I–sufficient RMA cells (C) or MHC-I–deficient RMA.ΔTAP2 cells (D) were s.c. injected and survival was monitored as in B. Both panels are cumulative of two independent experiments with 5–13 mice per group and statistics are from Log-rank (Mantel-Cox) test. **(E)** RMA.ΔTAP2.YFP cells were injected i.v. After 14 d, lungs were harvested and metastases were quantified using flow cytometry. Cumulative of two independent experiments with 5–12 mice per group. Statistics represent unpaired *t* test. **(F–H)** The number (F) and phenotype (G and H) of tumor-infiltrating NK cells in mice challenged s.c. with RMA.ΔTAP2 cells was analyzed by flow cytometry at day 14–21 after challenge. Cumulative of two independent experiments with eight to nine mice per group. Statistics represent unpaired *t* tests. Panels G and H were corrected for multiple testing using Bonferroni-Dunn. Error bars indicate SEM; ns, not significant; *P < 0.05, and ****P < 0.0001.

From the cell cycle–associated genes identified as downregulated in HuR-deficient NK cells (Fig. 4), we analyzed *Cdk6*, *Cdkn1a*, *Bub1*, *Plk1*, *Ndc80*, and *Ska2* mRNA abundance by Taqman PCR (Fig. 5 A). Ska2 and Ndc80 are both part of the spindle and kinetochore complex involved in chromosome separation during the last stages of mitosis. Both *Ndc80* and *Ska2* were confirmed to display decreased expression in HuR-deficient compared with littermate NK cells isolated on day 3 after MCMV infection, with *Ska2* displaying the biggest decrease (Fig. 6 A).

**Figure S3.**
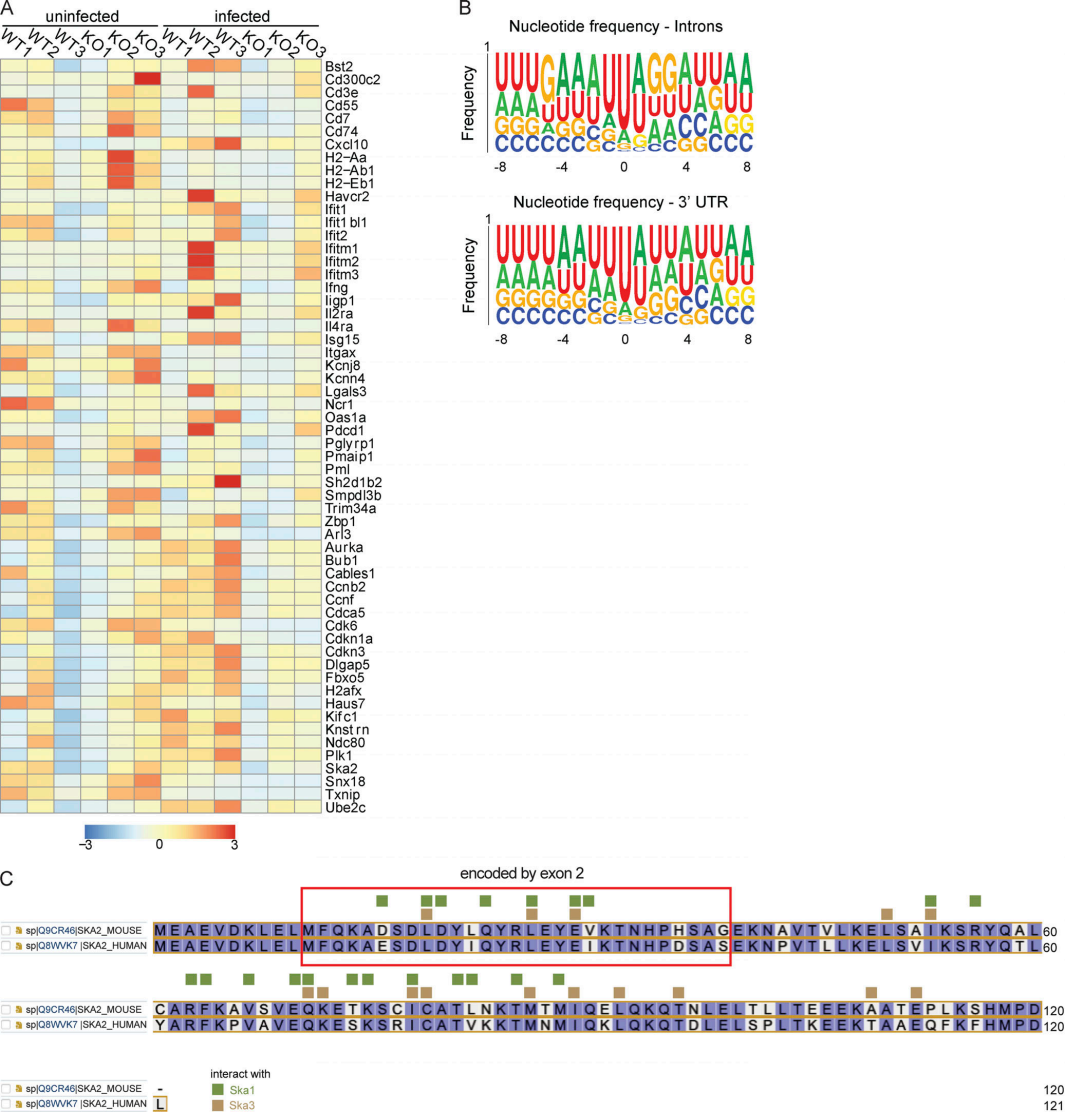
**Gene expression and HuR-binding consensus sequences in NK cells in response to MCMV infection and interactions of Ska2 with Ska1 and Ska3. (A)** Heatmap of differentially expressed genes associated with cell cycle and immune pathways. **(B)** Frequency plot for nucleotide residues found at HuR crosslinking sites in RNA, divided by introns and 3′UTRs. **(C)** Mouse and human SKA2 sequences were alined using UniProt (https://www.uniprot.org/align). The region of Ska2 that is encoded by exon 2 is marked by the red box. In green and yellow the amino acids that interact with Ska1 and Ska3, respectively, are indicated as published by Jeyaprakash et al. (2012).

Besides differential mRNA abundance, *Ska2* also displayed a significant increase in an alternatively spliced shorter isoform (excluding exon 2) in HuR-deficient NK cells (Fig. 5 B). This splice isoform produces a shorter protein without sites required for interactions with Ska1 and Ska3 within the kinetochore–microtubule complex (Jeyaprakash et al., 2012; Fig. S3). The Δexon2 isoform comprised ∼40% of all the transcripts in HuR CKO NK cells isolated from day 3 MCMV-infected animals, while isoforms lacking exon2 and 3 were not detected (Fig. 5 C). Sanger sequencing confirmed that exon 2 was specifically spliced out in the short isoform (Fig. 5 D), which encodes α-helices that interact with Ska1 and Ska3 (Fig. 5 E).

We next used CRISPR/Cas9 to target Ska2 and assess its role in primary NK cell expansion in vitro (Fig. 5, F and G). The established guide RNA (gRNA) targeting *Klb1c* (NK1.1; Riggan et al., 2020) reduced the surface expression of NK1.1 in ∼67% of NK cells, while the *Ska2* gRNA did not (Fig. 5 F). However, the *Ska2* gRNA impaired NK cell expansion in vitro by ∼85% as compared with *Klrbc* gRNAs, highlighting the essential role of Ska2 in NK cell expansion (Fig. 5 G). Thus, HuR-deficient NK cells display specific defects in gene expression and splicing associated with the spindle and kinetochore complex, particularly Ska2, which is essential for NK cell expansion.

### HuR is required in NK cells for the control of primary but not metastasizing tumors

To explore the role of HuR in NK cell–mediated tumor control, we first evaluated the ability of HuR CKO mice to control B16F10 metastases. To this end, we challenged NK cell–depleted HuR CKO or littermate controls with intravenously injected B16F10 cells. Lungs were harvested at 14 d after challenge and tumor nodules were enumerated in a blinded fashion. Lungs from NK cell–depleted mice contained high numbers of tumor nodules beyond the limit of quantitation (Fig. 6 A). Both HuR CKO and littermate controls eliminate most metastasizing B16F10 cells, and we did not observe significant differences between the two groups (Fig. 6 A). To investigate whether HuR impacts NK cell function in the context of solid tumors, we challenged NK cell–depleted, HuR CKO, and littermate control mice with B16F10 injected subcutaneously. All NK cell–depleted animals developed tumors and succumbed to the challenge within 30 d (Fig. 6 B and Fig. S5 A). While ∼40% of littermate controls were able to reject B16F10 tumors, none of the HuR CKO mice rejected the B16F10 tumors, indicating that HuR is required for NK cell–mediated control of solid tumors.

B16F10 melanoma is MHC-I sufficient and recognized through activation receptors (Lakshmikanth et al., 2009; Nagato et al., 2014). Certain tumors lose MHC-I during immunoediting (Dunn et al., 2004), which can be recognized by NK cells in a process termed “missing-self.” To investigate whether HuR is also involved in the control of MHC-I–deficient tumors, we generated a TAP2-deficient line (RMA.ΔTAP2) from a clonal MHC-I–sufficient RMA line using CRISPR (Fig. S5). We challenged PK136-treated, HuR CKO, and littermate control mice with subcutaneously injected RMA or RMA.ΔTAP2. As a negative control, all groups were unable to control the outgrowth of NK cell–insensitive RMA, and virtually all animals succumbed to the challenge by day 25 (Fig. 6 C and Fig. S5 B). In contrast, all littermate control animals were able to control RMA.ΔTAP2 tumors, while all PK136-treated and over 80% of HuR CKO animals succumbed to the tumor challenge (Fig. 6 D and Fig. S5 C), demonstrating that HuR is also required for NK cell–mediated control of MHC-I–deficient tumors. To investigate the impact of HuR-dependent NK cell expansion on control of MHC-I–deficient metastasizing tumors, we challenged HuR CKO and littermate control mice with Venus-YFP expressing RMA.ΔTAP2 intravenously. NK cell–depleted mice were not able to clear RMA.ΔTAP2, causing up to 50% of all lung CD45^+^ cells being tumor cells at 14 d after challenge (Fig. 6 E). Both HuR CKO and littermate controls virtually eliminated all metastasizing RMA.ΔTAP2 cells and no significant differences were between the two groups (Fig. 6 E). Thus, HuR CKO animals were defective in controlling two independent NK-reliant solid tumor models, while preserving the ability to clear metastasizing tumor cells of the same models.

### HuR CKO mice fail to accumulate NK cells in primary tumors

**Figure S4.**
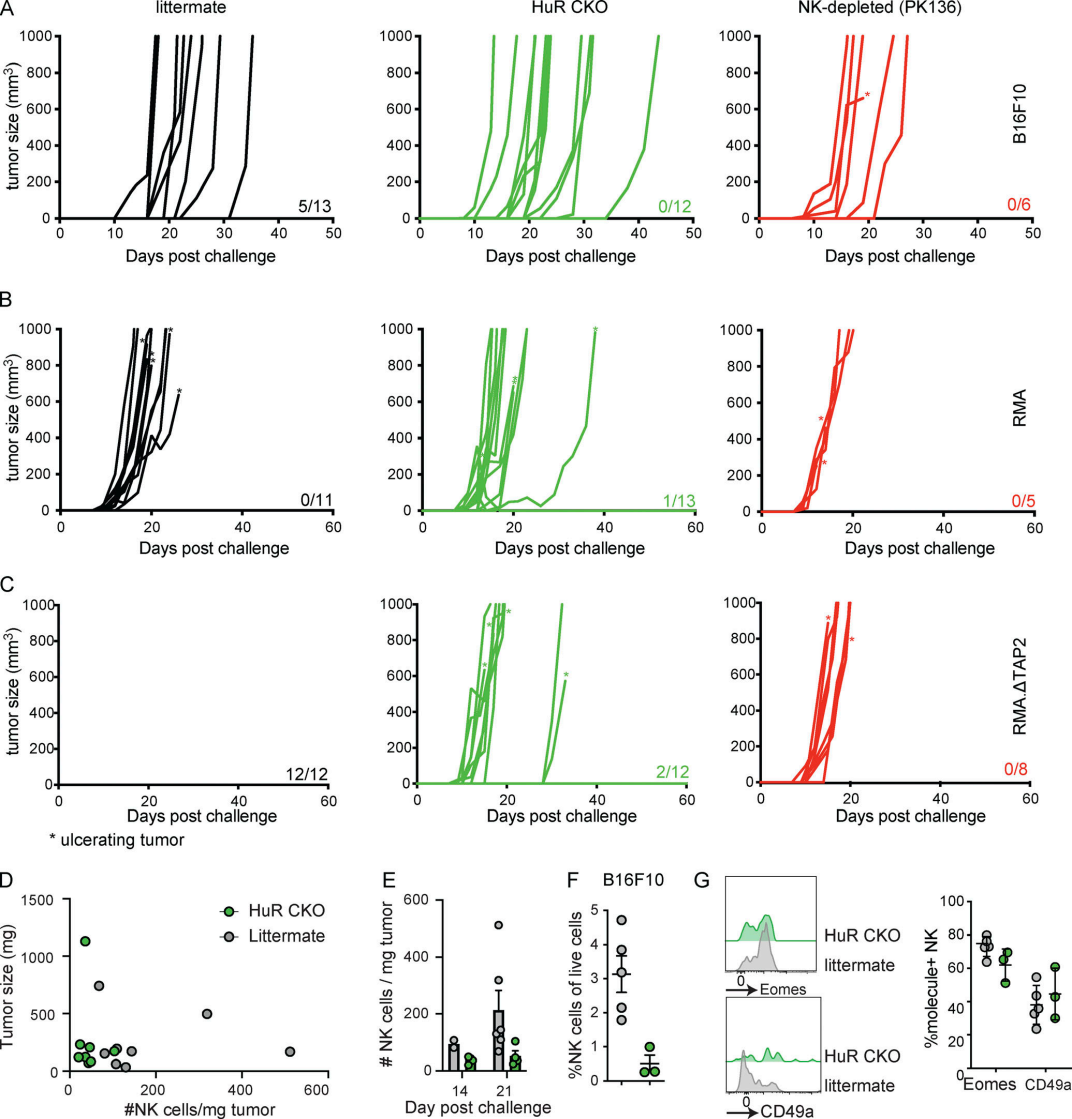
**Tumor outgrowth in HuR CKO mice. (A–C)** Tumor outgrowth in individual mice challenged s.c. with B16F10 (A), RMA (B), and RMA.ΔTAP2 (C). Above the x axis, the number of mice without a tumor at the end of the experiments versus the total mice in that group is indicated. Ulcerating tumors were indicated with * and were euthanized according to institutional guidelines. **(D and E)** The number versus tumor size (D) and kinetics (E) of tumor-infiltrating NK cells in mice challenged s.c. with 50,000 RMA.ΔTAP2 cells at day 14–21 after challenge. Cumulative of two independent experiments totaling eight to nine mice per group. **(F and G)** The number of tumor-infiltrating NK cells (F) and their phenotype (G) in indicated mice challenged with B16F10. Representative of two independent experiments with three to five mice per group.

**Figure S5.**
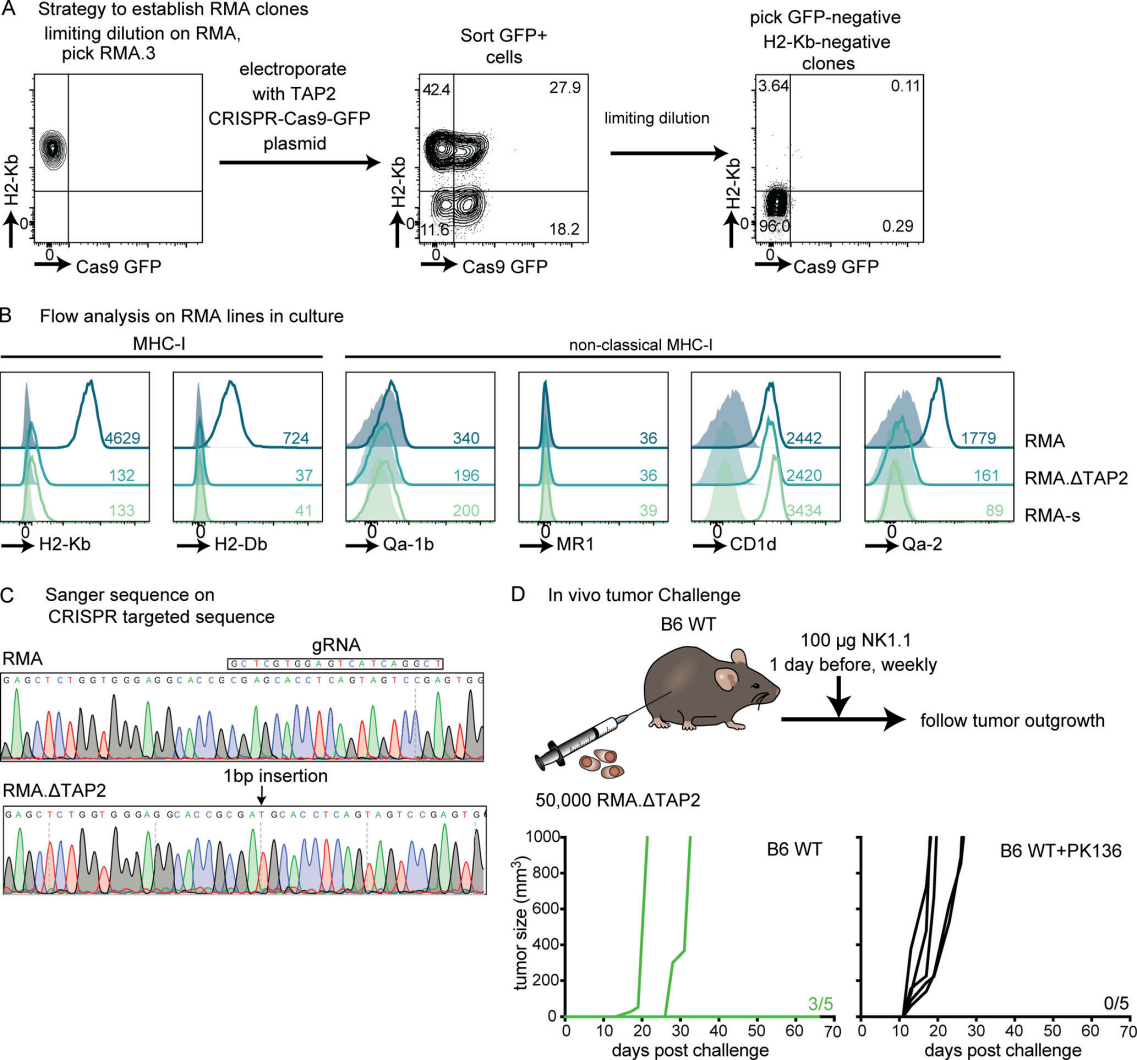
**Generation of TAP2-deficient RMA tumor model. (A)** Overview of RMA.ΔTAP2 tumor model generation. **(B)** Classical and non-classical MHC-I expression by tissue cultured RMA, RMA.ΔTAP2, and RMA-s using flow cytometry. **(C)** Sanger sequencing of genomic DNA TAP2 region targeted by CRISPR using primers forward 5′-CTT​TCC​GGT​GAA​CAA​GAA​GCC-3′ and reverse 5′-AAG​ATA​AGG​AGG​CTG​TGC​CC-3′. **(D)** 5 × 10^4^ of selected clone RMA.ΔTAP2 were s.c. injected and survival was monitored.

To evaluate defects of HuR-deficient NK cells to control primary tumors, we evaluated NK cell function and phenotype in tumor-infiltrating NK cells. HuR CKO and littermate controls were challenged with 5 × 10^4^ RMA.ΔTAP2 cells, a dose that causes tumor outgrowth in ∼50% of WT mice (Fig. S5 D). A fourfold reduction in the number of NK cells infiltrating RMA.ΔTAP2 tumors in HuR CKO mice was seen when compared with littermate controls (Fig. 6 F), which was not correlated with tumor size (Fig. S4 E) and was present at different time points after challenge (Fig. S4 F). HuR CKO NK cells showed a similar reduction in infiltration of B16F10 tumors (Fig. S4 G). HuR CKO NK cells infiltrating RMA.ΔTAP2 expressed similar levels of cytolytic molecules granzyme B and perforin as littermate controls (Fig. 6 G). Furthermore, we did not observe differences in expression of the markers PD-1 and KLRG1 (Fig. 6 G). While NK cells can convert into an ILC1-like phenotype within the tumor microenvironment (Gao et al., 2017), HuR CKO and littermate control NK cells displayed similar conversion into ILC1-like phenotype (Fig. 6 H). Thus, HuR is required for NK cell expansion within the tumor microenvironment, without overtly impacting NK cell phenotype. Taken together, our tumor data indicated that NK cell expansion is required for the control of solid tumors but is dispensable for NK cell–mediated elimination of metastasizing tumor cells.

## Discussion

Here, we found that the RNA-binding protein HuR serves a crucial function in NK cell expansion while being dispensable for other NK effector functions. This expansion deficiency in HuR CKO mice resulted in defective control of solid tumors and long-term virus infection. HuR-deficient NK cells displayed defects in the late phases of the cell cycle, the expression and splicing of cell cycle–associated genes, which was exemplified by *Ska2*, a component of the spindle and kinetochore complex. *Ska2* exhibited decreased expression and alternative splicing in the absence of HuR and was required for NK cell expansion in vitro. Thus, our findings uncovered a vital role for the posttranscriptional regulation of NK cell function by HuR, which regulated the cell cycle machinery required for NK cell expansion.

Our studies on HuR allowed us to evaluate the role of NK cell expansion, which we found is critical for the control of solid tumors and long-term virus infection without overtly affecting tumor cell metastasis and acute virus infection with MCMV. HuR-deficient NK cells displayed similar levels of Ki67 and BrdU incorporation compared with littermate controls, which represents active cell cycle and DNA replication during the S phase, respectively (Gratzner et al., 1975; Gerdes et al., 1984). However, HuR-deficient NK cells displayed defects in the G2/M phase, which was corroborated by transcriptome analysis revealing altered expression of the cell cycle genes *Ska2* and *Ndc80*. The Ska and Ndc80 complexes are essential in connecting chromosome kinetochores to microtubules allowing for correct chromosome separation during the metaphase of cell division (Nagpal and Fukagawa, 2016). The supramolecular Ska complex consists of 10 copies, each of Ska1, Ska2, and Ska3 (Jeyaprakash et al., 2012). We observed that in the absence of HuR, NK cells expressed lower levels of *Ska2* transcripts and ∼40% of them were of a shorter isoform that lacked exon 2. This shorter Ska2 isoform resulted in an in-frame deletion of 29 amino acids, including seven amino acids that interact with Ska1 and/or Ska3 (Jeyaprakash et al., 2012), thereby likely affecting the integrity of the Ska complex. The Ska complex interacts with the Ndc80 complex at the microtubule–kinetochore interphase (Huis In ’t Veld et al., 2019). The Ndc80 complex directly links the microtubule to the rest of the kinetochore and consists of Ndc80, Spc24, Spc25, and Nuf2 (Ciferri et al., 2005; Nagpal and Fukagawa, 2016). HuR-deficient NK cells also displayed lower expression of the Ndc80 complex component *Ndc80*, thereby further reducing the function of kinetochore machinery. Defects in the Ska and Ndc80 complexes likely result in mitotic catastrophe either in the metaphase before cytokinesis or in the G0/G1 phase soon after cytokinesis. Thus, HuR is required for proper NK cell expansion by affecting at least two independent complexes involved in the kinetochore during the metaphase of cell division, namely the Ska and Ndc80 complexes.

The HuR CKO NK cells lost HuR expression in stage 3 of NK cell development when NKp46 is expressed (Narni-Mancinelli et al., 2011), precluding any effects that HuR may play during NK cell development before this stage. Indeed, we did not observe substantial differences in NK cell numbers or NK cell maturation based on the markers CD27 and CD11b (Chiossone et al., 2009), indicating that NK cell development and maturation were chiefly unaffected in HuR CKO mice. In contrast, HuR has been implicated in T and B cell maturation and differentiation. In CD4^+^ T cells, HuR affects T helper activation and differentiation (Gubin et al., 2014; Chen et al., 2013), thereby contributing to asthmatic T helper 2 proinflammatory responses (Fattahi et al., 2022). We did not observe differences in cytokine production, even under conditions that are tightly regulated by transcription and translation (Piersma et al., 2019), potentially because NK cells do not require helper differentiation as CD4^+^ T cells do. In B cells, HuR was found to be essential for the B cell antibody and germinal center responses (Osma-Garcia et al., 2021; Diaz-Muñoz et al., 2015). LPS-stimulated B cells require HuR for metabolic fitness including alternative splicing of *dlst* (Diaz-Muñoz et al., 2015), which was not detected in germinal center B cells (Osma-Garcia et al., 2021). We did not observe evidence for differences in metabolic fitness in HuR-deficient NK cells. Germinal center B cells require HuR for Ig somatic hypermutation and associated DNA damage and cell death pathways (Osma-Garcia et al., 2021). These pathways did not play a role in HuR-deficient NK cells, consistent with the notion that NK cells are innate lymphocytes and do not employ somatic alterations. Instead, we primarily observed defects in the cell cycle, resulting in deficient NK cell expansion in response to virus infection and tumor challenge. These cell cycle defects may also play a role in other lymphocyte lineages but have not been previously described, possibly because they are potentially obscured (in part) by other cell-specific processes that are mediated by HuR.

Prior studies on the role of NK cell expansion during viral infection have been somewhat difficult to reconcile. NK cell recognition of m157-expressing cells through Ly49H results in absolute protection from MCMV infection in C57BL/6 mice in terms of survival and around a 1,000-fold reduction in viral load (Scalzo et al., 1992; Brown et al., 2001). This protection is mainly mediated through the cytolytic activity of NK cells as it is abrogated with genetic deficiencies in molecules required for the cytolytic machinery such as Unc13d, granzyme B, and perforin (Crozat et al., 2007; Fehniger et al., 2007), which in turn are specifically mediated through Ly49H-dependent NK cell recognition of m157 (Parikh et al., 2015). NK cell–mediated, Ly49H-dependent elimination of MCMV in spleen is evident by day 2–3 p.i. (Loh et al., 2005), before Ly49H^+^ NK cell expansion, which is detectable around day 4 p.i. and peaks at day 7 p.i. after which it contracts to form a long-lived adaptive compartment (Dokun et al., 2001; French et al., 2006; Sun et al., 2009), indicating the majority of NK cell expansion starts after the virus is acutely controlled, unlike T cell clonal expansion that occurs prior to T cell viral control (Wherry and Ahmed, 2004; Wherry et al., 2003). Consistent with these observations, mice with NK cell–expansion defects display only modest defects in acute MCMV control, though these studies also show other effects on NK cell functions, making it difficult to determine the effect of NK cell expansion defects (Adams et al., 2018; Dong et al., 2019; Mah-Som et al., 2021; Victorino et al., 2021). Here, we show that despite the inability of HuR-deficient NK cells to expand, there were no overt defects in control of MCMV infection with respect to morbidity and viral load in HuR CKO animals in a range of organs as compared with littermate controls under the conditions tested. Recent data from our group has indicated that CD8^+^ T cells can impact MCMV control as early as 4 d p.i. (Parikh et al., 2020), which could potentially compensate for partial loss of NK cell–mediated MCMV control. To exclude such T cell compensation, we evaluated RAG HuR CKO mice and observed that these mice had no defects in acute viral control, weight loss, or survival compared with littermate controls, providing further evidence that NK cell expansion is dispensable for acute MCMV control. Yet, we observed accelerated weight loss and increased viral load at later time points after infection in RAG HuR CKO animals, indicating that HuR-dependent NK cell expansion is required for long-term viral control in the absence of adaptive immunity. Consistent with these findings, RAG KO animals treated with broad-spectrum antibiotics displayed increased long-term MCMV control, which was associated with increased NK cell expansion (Kamimura and Lanier, 2015). Thus, our results reveal that even though NK cell expansion has minimal effect on acute viral control, it contributes to long-term viral control in specific organs, particularly when functional adaptive immunity is absent.

These findings have further implications for patients with genetic defects in general cell division that present with a predisposition to virus infections (Gineau et al., 2012; Cottineau et al., 2017; Mace et al., 2020; Hanna et al., 2015). These patients might be able to control virus infections during acute phase of viral infections but manifest defective NK cell control in later phases of viral infection, as suggested by our mouse studies. This expansion-dependent phenomenon may also apply to NK cell–mediated solid tumor control in humans as well.

NK cells are potent in eradicating certain tumor metastases, including circulating B16 melanoma metastases in C57BL/6 mice (Gorelik et al., 1982). Besides eradicating B16 metastases, NK cells also contribute to controlling B16 melanoma in the context of solid tumors (van Elsas et al., 1999). Control of B16 lung metastases has recently been reported to mainly occur within the first 24 h after inoculation (Ichise et al., 2022), suggesting early control of metastases is unlikely to require expansion of NK cells. Consistent with this premise, HuR CKO animals that were defective in NK cell expansion were capable of eradicating B16 tumor metastases with no significant differences compared with littermate controls. In contrast, HuR CKO animals were significantly impaired in controlling B16 solid tumors, indicating that NK cell expansion is required for optimal control of solid tumors.

Tumors can evade T cell recognition by acquiring mutations in the MHC-I pathway, which appears to be a tumor-escape pathway in patients who relapse after immunotherapy (Schoenfeld and Hellmann, 2020). These escaped tumors are potentially recognized by NK cells through missing-self, which does not appear to be effective in these patients (Malmberg et al., 2017). HuR CKO mice were unable to control TAP2-deficient tumors, indicating that HuR-dependent NK cell expansion is vital for NK cell–dependent control of MHC-I–deficient tumors.

Interestingly, MHC-I loss is common in primary colorectal tumors, yet colorectal liver metastases are MHC-I positive (Anderson et al., 2021). These observations suggest that NK cells efficiently eliminate MHC-I–deficient colorectal tumor cells in circulation before establishing liver metastases while being inefficient in clearing MHC-I–deficient tumor cells within the primary tumor. This theory is further supported by our observations that HuR-deficient NK cells are fully capable of controlling (MHC-I deficient) metastases but are defective in controlling subcutaneous tumors. These findings furthermore imply that even under conditions where NK cells can eliminate metastasizing tumor cells, they may be inefficient in eliminating solid tumors within the tumor microenvironment. Boosting NK cell expansion, in particular during instances where NK cell numbers are low, may increase effectiveness of NK cells in solid malignancies.

Finally, our studies further highlight fundamental differences and similarities between NK cells and T cells in terms of their immune responses. An expansion of NK cells during initial phases of immune responses may be dispensable, unlike for T cells. However, like T cells, an expanded NK cell responding population is needed for optimal viral control in later phases and is necessary for certain antitumor responses, particularly against primary solid tumors.

## Materials and methods

### Mice

C57BL/6 (stock number 665) mice were purchased from Charles River Laboratories. *Elavl1*^*fl/fl*^ mice (021431; Ghosh et al., 2009), *Rag1* KO (002216; Mombaerts et al., 1992), and CD45.1 (002014) were purchased from Jackson Laboratories. Rag1. *Ncr1*^*Cre*^ mice (Narni-Mancinelli et al., 2011) were kindly provided by Eric Vivier, Aix Marseille University, Marseille, France. B6.BxD8 mice (Cheng et al., 2008) were generated and maintained in our laboratory. *Rag2*^*−/−*^*Il2rg*^*−/−*^ mice were generated by crossing *Rag2*^*−/−*^ mice with *Il2rg*^*−/−*^ mice as previously described (Park et al., 2019). Age- and sex-matched mice were used in all experiments. Potential germline deletion of *Elavl1* was analyzed using primers WT-forward 5′-CTC​TCC​AGG​CAG​ATG​AGC​A-3′, deletion-forward 5′-TCT​GGG​TCC​TTA​GCA​TAT​GAG​G-3′, and common-reverse 5′-TAG​GCT​CTG​GGA​TGA​AAC​CT-3′ with an annealing temperature (Tm) of 60°C for 35 cycles using DreamTAQ polymerase (Thermo Fisher Scientific) according to the manufacturer’s instructions. Mice with germline deletions (<1/200) were excluded from experiments and breeding. All mouse studies were conducted in accordance with Washington University institutional ethical guidelines through institutional animal care and use committee protocol that was approved by the Animal Studies Committee under protocol number 21–0090.

### MCMV infection

Where indicated, mice were infected i.p. with 2 × 10^4^ PFU salivary gland WT1 MCMV as previously described (Piersma et al., 2020). For morbidity and survival studies, weights were monitored every other day and mice were sacrificed when more than 30% of the initial weight was lost, in accordance with institutional guidelines. Viral load analysis was performed as previously described (Parikh et al., 2015). Briefly, RNA-free organ DNA was isolated using Puregene extraction kit (Qiagen), and brain and lung DNA was cleaned with phenol–chloroform–isoamyl alcohol. DNA was quantified for MCMV IE1 (forward: 5′-CCC​TCT​CCT​AAC​TCT​CCC​TTT-3′; reverse: 5′-TGG​TGC​TCT​TTT​CCC​GTG-3′; probe: 5′-TCT​CTT​GCC​CCG​TCC​TGA​AAA​CC-3′; IDT DNA) and host *Actb* (forward: 5′-AGC​TCA​TTG​TAG​AAG​GTG​TGG-3′; reverse: 5′-GGT​GGG​AAT​GGG​TCA​GAA​G-3′; probe: 5′-TTC​AGG​GTC​AGG​ATA​CCT​CTC​TTG​CT-3′; IDT DNA) against plasmid standard curves using TAQman universal master mix II on a StepOnePlus real time PCR system (Thermo Fisher Scientific).

At indicated times, lymphocytes were isolated from blood, the spleen, and/or the liver. EDTA blood was collected, and red blood cells were lysed with Tris-NH_4_Cl RBC lysis buffer. Spleens were homogenized using cell strainers and treated with RBC lysis buffer to obtain single-cell splenocyte solutions. Livers were homogenized using cell strainers and hepatocytes were removed with 39% isotonic percoll and treated with RBC lysis buffer.

BrdU incorporation assays were performed as previously reported (French et al., 2006); 5 d p.i., mice were pulsed with 2 mg BrdU for 3 h before analysis by flow cytometry. For transfer studies into B6.BxD8 mice, a mixture of HuR CKO or littermate control splenocytes were mixed with congenic C57BL/6 splenocytes and i.v. injected into B6.BxD8 mice. The next day mice were bled and subsequently infected i.p. with 5 × 10^3^ PFU WT1 MCMV. Mice were bled at indicated time points and Ly49H expansion was analyzed by flow cytometry.

For analysis of m157 mutants, splenic DNA was amplified for m157 using the primers forward 5′-CAT​AAT​TCC​CAT​CGT​CAC​TAG​AG-3′ and reverse 5′-CAT​AAT​TCC​CAT​CGT​CAC​TAG​AG-3′ using Phusion (Thermo Fisher Scientific) in HF buffer with Tm 64°C for 25 cycles according to the manufacturer’s instructions, and the product was cloned into pCR-Blunt-II-TOPO and m157 sequences of 10 colonies per mouse were analyzed using Sanger sequencing.

### Adoptive transfer into Rag2^−/−^Il2rg^−/−^

HuR CKO or littermate control NK cells were purified by negative selection (Stem Cell Technologies) to a purity of 88–91%. 200,000 purified NK cells were i.v. injected into *Rag2*^−*/*−^*Il2rg*^−*/*−^ mice, and 14 d after transfer, liver NK cells were analyzed by flow cytometry.

### In vitro stimulation assays

Stimulation of NK cells with m157-Tg murine embryonic fibroblasts in the presence/absence of IL-12 (PeproTech) was performed as previously described (Piersma et al., 2019; Parikh et al., 2015). NK1.1-dependent NK cell expansion was performed in concordance with previous studies (Reichlin and Yokoyama, 1998). Briefly, NK cells were purified to 50–80% purity by negative selection (Stem Cell Technologies), and 40,000 purified NK cells were added to anti-NK1.1-coated (4 µg/ml) 96-well flat-bottom plate and cultured in the presence of 100 U/ml IL-2 for 4 d. For cytokine-dependent NK cell expansion, 40,000 purified NK cells were cultured with 1,000 U/ml IL-2 or 100 ng/ml IL-15 (PeproTech) for 4 d and analyzed by flow cytometry.

### Flow cytometry and cell sorting

Fluorescent-labeled antibodies CD107a (clone eBio1D4B), CD11b (M1/70), CD19 (eBio1D3), CD27 (LG.7F9), CD3 (145-2C11), CD4 (RM4-5), CD45 (30-F11), CD45.1 (A20), CD45.2 (104), CD69 (H1.2F3), CD8 (53-6.7), CD94 (18d3), Eomes (Dan11mag), granzyme B (GB12), IFNγ (XMG1.2), Ki67 (SolA15), KLRG1 (2F1), Ly49D (4D11), Ly49E/F (CM4), Ly49G2 (eBio4D11), Ly49I (YLI-90), Ly6C (HK1.4), NKG2A/C/E (20D5), NKG2AB6 (16a11), NKp46 (29A1.4), and TCRB (H57-597) were purchased from Thermo Fisher Scientific; CD3 (17A2), Ly49H (3D10), NK1.1 (PK136), perforin (S16009B), and PD1 (29F.1A12) were purchased from BioLegend; CD49a (Ha31/8), Ly49A (A1), and Ly49F (HBF-719) were purchased from BD Bioscience; HuR (3A2) was purchased from Santacruz; and Ly49C (4LO) was produced in-house.

For quantification of cell numbers, 5,000 Precision Count Beads (BioLegend) were added before staining. Cells were subsequently stained with fixable viability day (Thermo Fisher Scientific), continued by staining of cell surface molecules in 2.4G2 hybridoma supernatant to block Fc receptors. For intracellular staining, cells were fixed and stained intracellularly using the eBioscience Foxp3/Transcription Factor Staining Buffer Set (Thermo Fisher Scientific) according to the manufacturer’s instructions. BrDU (BD Bioscience) and caspase3/7 plus Sytox-AADvanced (Thermo Fisher Scientific) were stained according to the manufacturer’s instructions. Samples were acquired using FACSCanto (BD Biosciences) and analyzed using FlowJo software (BD Biosciences). NK cells were defined as Singlets Viability-NK1.1^+^NKp46^+^CD3^−^CD19^−^ in peripheral blood, spleen, and tumor, and Singlets Viability-NK1.1^+^NKp46^+^Eomes^+^CD49a^−^CD3^−^CD19^−^ in the liver. Where indicated, cells were sorted on a FACSAria (BD Biosciences) into media and subsequently lysed in Trizol for RNA analysis or RNA crosslinking.

### RNA-seq analysis

NK cells (NK1.1^+^) were sorted from naïve or day 3 MCMV-infected spleens from HuR CKO or littermate controls. For MCMV conditions, three mice were pooled per sample. RNA was isolated from sorted NK cells using Trizol (Thermo Fisher Scientific). Downstream RNA-seq and analysis were performed as previously described (Bangru et al., 2018). Briefly, RNA quality was assessed, Hi-Seq libraries were prepared, and 100-bp paired-end Illumina sequencing was performed on a HiSeq 4000. RNA-seq reads were processed for quality and read length filters using Trimmomatic (version 0.38) and were aligned to the mouse genome (mm10) using STAR (version 2.4.2a). Gene expression levels were defined as transcripts per million using count and differential expression values obtained from DESeq2 (version 1.8.2) and HTseq (version 0.6.1). Genes were considered as having significant differential expression following imposed cutoff clearance (false discovery rate [FDR] < 0.05, log_2_[fold change] > 1). Differential splicing analysis was performed using rMATS (version 3.2.5), and significant events were identified using imposed cutoffs (FDR < 0.10, junction read counts ≥10, percent spliced in ≥15%). Gene ontology analysis was performed using DAVID (version 6.8) and mapped using the “Enrichment Maps” plugin in Cytoscape. All expressed genes with transcripts per million >1 served as background and the biological function category was analyzed with three pathways (Biocarta, Kegg, and Panther). Functional clustering was executed and the top clusters (P value <0.05) were represented. For grouping genes into clusters, we first overlapped the differentially expressed gene sets (FDR < 0.05, log_2_ [fold change] > 1) between uninfected (littermate) and MCMV-infected (littermate) categories. The overlapping and non-overlapping gene sets were then grouped and their corresponding average fold change relative to MCMV-infected (littermate) was calculated and plotted.

### eCLIP library preparation

HuR eCLIP was performed in concordance with previously published protocols (Van Nostrand et al., 2016). In short, NK cells were sorted from uninfected or day 3 MCMV-infected C57BL/6 spleens. For uninfected samples, spleens from 10 mice were pooled, and for MCMV-infected samples, spleens from 20 mice were pooled. UV crosslinking was performed with 1 pulse of 400 mJ/cm^2^. Briefly, crosslinked cells were lysed in buffer and sonicated, followed by treatment with RNase I (Thermo Fisher Scientific) to fragment RNA. HuR antibody (3A2; Santacruz) was precoupled to anti-mouse IgG Dynabeads (11201D; Thermo Fisher Scientific), added to lysate, and incubated 3 h at 4°C. Prior to IP washes, 2% of sample was removed to serve as the paired input sample. For IP samples, high- and low-salt washes were performed, after which RNA was dephosphorylated with FastAP (Thermo Fisher Scientific) and T4 PNK (NEB) at low pH, and a 3′ RNA adaptor was ligated with T4 RNA ligase (NEB). 15% of IP and input samples were run on an analytical 4–12% PAGE gel, transferred to polyvinylidene fluoride membrane, blocked in 5% dry milk in tris-buffered saline with Tween, incubated with anti-HuR antibody, washed, incubated with HRP-conjugated anti-mouse secondary (1706516; Bio-Rad), and visualized with chemiluminescence imaging to validate successful IP. The remaining IP and input samples were run on a 4–12% PAGE gel and transferred to nitrocellulose membranes, after which the region from the protein size to 75 kD above protein size was excised from the membrane, treated with proteinase K (NEB) to release RNA, and concentrated by column purification (Zymo). Input samples were then dephosphorylated with FastAP (Thermo Fisher Scientific) and T4 PNK (NEB) at low pH, and a 3′ RNA adaptor was ligated with T4 RNA ligase (NEB) to synchronize with IP samples. Reverse transcription was then performed with AffinityScript (Agilent), followed by ExoSAP-IT (Affymetrix) treatment to remove unincorporated primer. RNA was then degraded by alkaline hydrolysis, and a 3′ DNA adaptor was ligated with T4 RNA ligase (NEB). qPCR was then used to determine the required amplification, followed by PCR with Q5 (NEB) and gel electrophoresis to size-select the final library. Libraries were sequenced on the NovaSeq6000 platform (Illumina). eCLIP was performed on IP from two independent samples along with paired size-matched input before the IP washes.

### RNA expression analysis

RNA was isolated from sorted cells using Trizol according to the manufacturer’s instruction (Thermo Fisher Scientific). Complementary DNA was synthesized from Turbo DNAse-treated using Superscript III with oligo(dT) (Thermo Fisher Scientific). Quantification was performed with primer/probes for *Elavl1* (Mm.PT.58.31692504), *Elavl2* (Mm.PT.58.8201242), *Elavl3* (Mm.PT.58.30335452), *Elavl4* (Mm.PT.58.33020194), *Cdk6* (Mm.PT.58.7685465), *Cdkn1a* (Mm.PT.58.5884610), *Bub1* (Mm.PT.58.5537162), *Plk1* (Mm.PT.58.12563595), *Ndc80* (Mm.PT.56a.21711652), and *Ska2* (Mm.PT.56a.9869139; all from IDT DNA) and normalized to *Gapdh* (Mm99999915_g1; Thermo Fisher Scientific) against plasmid or geneblock (IDT DNA) standard curves using TAQman universal master mix II on a StepOnePlus real-time PCR system (Thermo Fisher Scientific).

Alternative splicing of Ska2 was analyzed with forward 5′-GAG​GTC​GAT​AAG​CTG​GAA​CTG​A-3′ and reverse 5′-CTC​TAG​ACG​TCT​CGC​CCA​AT-3′ primers located on exon1 and exon4, respectively, using DreamTAQ polymerase (Thermo Fisher Scientific) with a Tm of 60°C for 35 cycles according to the manufacturer’s instructions. After resolving on 1.5% agar gel, bands were quantified using Image Lab (Bio-Rad). Indicated bands were excised and gel-extracted (Macherey-Nagel) followed by Sanger sequencing (Genewiz).

### CRISPR in primary NK cells

NK cells were edited with CRISPR as previously described (Riggan et al., 2020). Briefly, splenocytes were cultured with 50 ng/ml IL-15 overnight and electroporated with Cas9 complexed with NK1.1 (5′-GAG​GAA​GGT​CAA​GCT​GAC​TG-3′; Riggan et al., 2020) or Ska2 (5′-GGT​CGA​TAA​GCT​GGA​ACT​GA-3′) gRNA selected using CRISPick (Doench et al., 2016; all from Synthego) using Neon transfection (Thermo Fisher Scientific). Electroporated splenocytes were cultured for 4 d in the presence of 50 ng/ml IL-15 and NK cells were subsequently analyzed by flow cytometry.

### Tumor studies

The tumor cell line RMA-s is a prototypic tumor cell line that is recognized through missing-self because of a point mutation in TAP2 (Kärre et al., 1986; Yang et al., 1992). However, reconstitution of TAP2 does not fully revert tumor outgrowth as compared with the parental RMA line (Franksson et al., 1993). To circumvent TAP2-independent recognition by NK cells, we generated TAP2-deficient RMA cells using CRISPR (Fig. S5). A clone from RMA, isolated using limiting dilution, was used as RMA in this manuscript. This clone was subsequently electroporated with SpCas9-2A-GFP plasmid (48138; Addgene; Ran et al., 2013) containing the gRNA sequence 5′-TCG​GAC​TAC​TGA​GGT​GCT​CG-3′ targeting *Tap2* that was selected using CRISPick (Doench et al., 2016). GFP-positive cells were sorted using FACSAria 2 d after transfection, 2 wk after sort H-2K^b^-negative and GFP-negative cells were sorted to circumvent effects of CAS9, and clones were isolated by limiting dilution. The selected clone was further characterized for surface markers and in vivo tumor growth; the clone used in these studies was designated RMA.ΔTAP2. This clone was subsequently transduced with YFP-AKAluciferase retrovirus to track tumor spread in vivo.

B16F10 metastasis studies were performed as previously described (Overwijk and Restifo, 2001). Briefly, 3 × 10^5^ B16F10 cells (ATCC) were injected i.v. into indicated mice. For NK-depletion controls, mice were administered i.p. with 100 μg anti-NK1.1 antibody (clone PK136), 2 d before and 5 d after tumor cell inoculation. 2 wk after challenge, lungs were harvested, perfused with PBS, and fixed with Feteke’s solution. Tumor nodules were counted using a dissecting microscope in a blinded fashion. Lungs with more than 500 nodules were considered above the limit of detection. For RMA.ΔTAP2 metastases studies, 5 × 10^4^ RMA.ΔTAP2.YFP cells were injected i.v. into indicated mice. 2 wk after challenge, lungs were harvested, digested with 1 mg/ml Collagenase Ia (Millipore Sigma) and 400 µg/ml DNase I (Millipore Sigma), and analyzed by flow cytometry.

For solid tumor studies, 10^5^ B16F10, 10^4^ RMA, or 10^4^ RMA.ΔTAP2 cells were subcutaneously (s.c.) injected in the flank in 200 µl PBS. Where indicated, mice were depleted with anti-NK1.1 (PK136) 2 d before tumor challenge, followed by weekly depletions. Tumor sizes were measured three times per week with a caliper and mice were sacrificed when tumors reached sizes >1,000 mm^3^, in accordance with ethical guidelines. For analysis of tumor-infiltrating lymphocytes, mice were challenged with 10^5^ B16F10 or 5 × 10^4^ RMA.ΔTAP2 s.c. in the flank. At the indicated day after challenge, tumors were dissected and digested with Collagenase Ia and DNase I followed by flow cytometric analysis.

### Statistical analysis

Statistical analysis was performed with Prism (GraphPad software) using Log-rank (Mantel-Cox), unpaired *t* tests, and two-way ANOVA as indicated in the figure legends. Error bars in figures represent the SEM. Statistical significance was indicated as follows: ****P < 0.0001; ***P < 0.001; **P < 0.01; and *P < 0.05; ns, not significant.

### Online supplemental material

Fig. S1 shows HuR expression in NK cells and phenotyping at steady state. Fig. S2 shows HuR expression in response to MCMV infection and additional analysis related to Fig. 1 and Fig. 2. Fig. S3 shows additional RNA-seq analysis related to Fig. 4 and interactions of Ska2 with other family members related to Fig. 5. Fig. S4 shows tumor growth in individual mice and additional analysis related to Fig. 6. Fig. S5 shows development of the RMA.ΔTAP2 model related to Fig. 6.

## Supplementary Material

SourceData F5 is the source file for Fig. 5.

## Data Availability

The data underlying the manuscript are available in the published article and its online supplemental material. All raw sequencing data files are available for download from the Gene Expression Omnibus under accession GSE217392. Materials generated in these studies are available upon request via material transfer agreement with Washington University in St. Louis.
