# Supplementary material for: NK cell expansion requires HuR and mediates control of solid tumors and long-term virus infection
Source: J Exp Med. 2023 Sep 12;220(11):e20231154. doi: 10.1084/jem.20231154 (PMC10497399; doi:10.1084/jem.20231154)
Supplement: SourceData F5 — is the source file for Fig. 5. [file JEM_20231154_SourceDataF5.pdf]

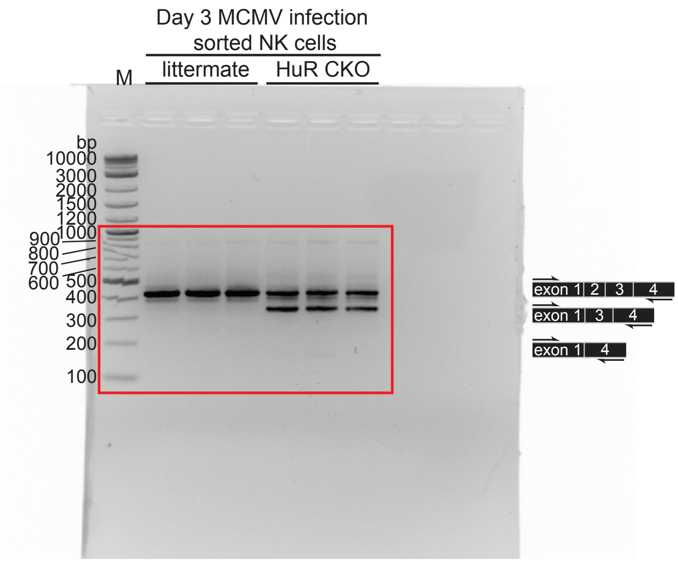


the gel broke during transfer to imager causing
an artefact in the marker lane (M) containing
Purple 1 kb Plus DNA ladder from New England BioLabs
